# Tetraalkyl Hydroxymethylene-bisphosphonate and Dialkyl 1-Diphenylphosphinoyl-1-hydroxy-ethylphosphonate Derivatives by the Pudovik Reaction and Their Rearranged Products

**DOI:** 10.3390/molecules26247575

**Published:** 2021-12-14

**Authors:** Zsuzsanna Szalai, György Keglevich

**Affiliations:** Department of Organic Chemistry and Technology, Budapest University of Technology and Economics, 1521 Budapest, Hungary; sz.zsuzsi97@gmail.com

**Keywords:** α-oxophosphonate, dialkyl phosphite, diphenylphosphine oxide, Pudovik reaction, hydroxymethylene-bisphosphonate, 1-phosphinoyl-1-hydroxy-ethylphosphonate, rearrangement

## Abstract

The reaction of diethyl α-oxoethylphosphonate and diethyl oxobenzylphosphonate with diethyl phosphite, dimethyl phosphite, and diphenylphosphine oxide affords, depending on the substrates and conditions (nature and quantity of the amine catalyst, temperature, and solvent), the Pudovik adduct and/or the corresponding >P(O)–CH–O–P(O)< product formed by rearrangement. The nature of the substituent on the central carbon atom (a methyl or phenyl group) influences the inclination for the rearrangement. The asymmetric products (either adducts or rearranged species) with different P(O)Y functions (Y = RO or Ph) exhibit interesting NMR features.

## 1. Introduction

Hydroxymethylene bisphosphonic (dronic) acid derivatives are of importance due to their beneficial effect in the treatment of bone diseases [1,2,3,4]. Their synthesis, starting from the corresponding substituted acetic acid and phosphorus trichloride/phosphorous acid, was thoroughly investigated [1,5,6]. Another approach is the Pudovik reaction (vide infra), the original version of which involves the addition of dialkyl phosphites onto the carbonyl carbon of oxo compounds, such as aldehydes and ketones [7,8].

**Figure 1:**
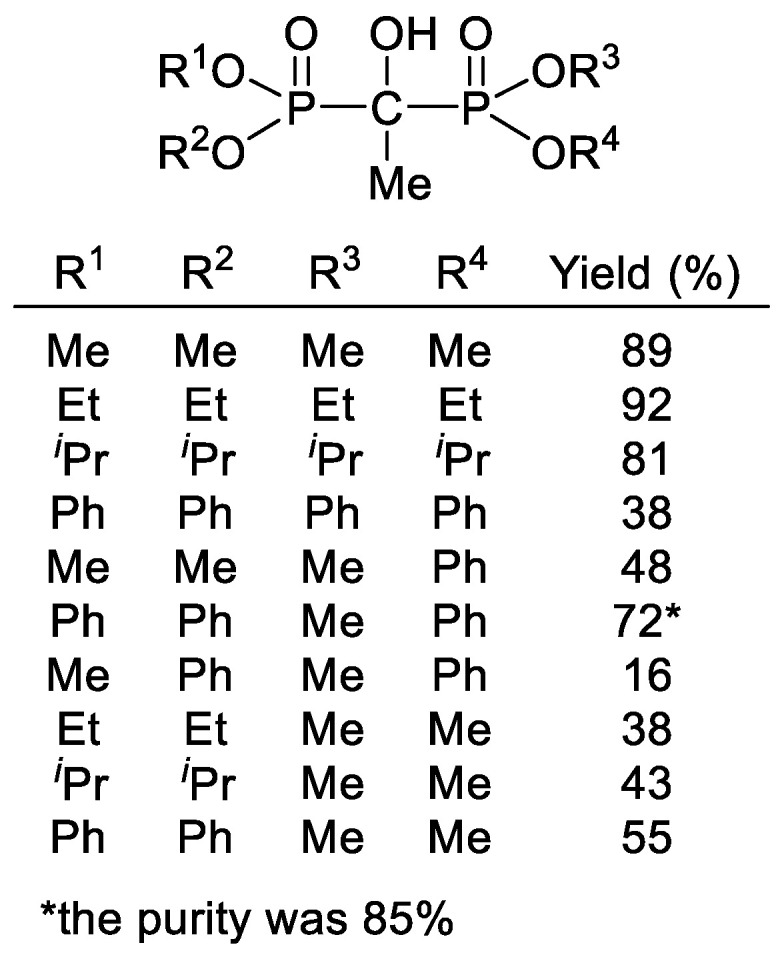
1-Hydroxyethylidene-1,1-bisphosphonates synthesized by Vepsäläinen et al.

In 1956 McConnell and Coover described that the solvent-free reaction of diethyl α-oxoethylphosphonate with diethyl phosphite using diethylamine as the catalyst afforded the adduct tetraethyl α-hydroxy-α-ethylidenebisphosphonate [9]. The product was claimed to be identified by boiling point, refractive index, elemental analysis and IR spectral data. Six years later, Fitch and Moedritzer proved that the corresponding phosphonate-phosphate was formed in the above experiment [10]. The principal proof was the ^31^P-NMR spectrum, as the species under discussion exhibited two δ_P_ shifts at −1.3 and 20.2 Nicholson and Vaughn prepared an analogous oxophosphonate–dimethyl phosphite adduct at 0 °C using diethyl ether as the solvent, and 0.05 equivalents of dibutylamine as the catalyst [11]. After thirty years, Vepsäläinen and Turhanen and their co-workers prepared a series of symmetrical and asymmetrical tetraalkyl 1-hydroxyethylidene-1,1-bisphosphonates, making use of the Nicholson method. The products obtained in variable yields (16–92%) are shown in Figure 1. There was no mention of the rearrangement [12]. A few tri-, P,P′-di-, P,P-di-, and mono esters of 1-hydroxyethylidene-bisphosphonic derivatives were also described [13].

Later on, both the diethyl α-oxoethylphosphonate–diethyl phosphite adduct and the related rearranged product could be selectively prepared in a solvent-free microwave-assisted manner, by applying a temperature of 120 °C for 20 min together with 5% of diethylamine (or dibutylamine) catalyst, or 50% dibutylamine additive, respectively [14,15]. Hammerschmidt and co-workers investigated the stereochemical course of the α-hydroxyphosphonate–phosphate rearrangement in general, and they proved that this transformation proceeds with the retention of configuration [16,17].

It was a challenge for us to investigate the possibilities for the fine tuning of this reaction, and to expand its scope by synthesizing mixed derivatives comprising the (EtO)_2_P(O)CZ(OH)P(O)Y_2_ scaffold, where Y = alkoxy or Ph and Z = Me or Ph.

## 2. Results and Discussion

In the first case, diethyl α-oxoethylphosphonate obtained by the Arbuzov reaction of acetyl chloride and triethyl phosphite, was subjected to the Pudovik reaction with diethyl phosphite in the presence of diethylamine, or dibutylamine under different conditions (Table 1). Besides the expected tetraethyl α-hydroxy-methylphosphonate (**2**), the rearranged product (**3**) was also formed. Performing the addition in diethyl ether at 0 °C for 8 h and applying Et_2_NH in quantities of 5%, 20% and 40%, the ratio of products **2** and **3** was 100–0, 98–2 and 87–13, respectively (Table 1, entries 1–3). Bu_2_NH was also a suitable catalyst: in the presence of 5% amine, the adduct **2** was obtained in a selectivity of 99% (Table 1, entry 4). Then, in the hope of shifting the product ratio towards favoring the rearranged species **3**, we carried out the reactions under solvent-free conditions at higher temperatures. Applying 40% Et_2_NH at 120 °C and 135 °C for 20 min, the ratio of species **2** and **3** was 66–34 and 51–49, respectively (Table 1, entries 5 and 6). On further heating at 135 °C for 3 h, the ratio of the two components (**2** and **3**) practically reversed (66–34% changed to 32–68%) (Table 1, entry 5 and footnote “b”). Bu_2_NH (5%) remained selective with respect to the addition, as after a reaction at 120 °C for 20 min, the ratio of products **2** and **3** was 94–6 (Table 1, entry 7). In order to shift the ratio in favor of the rearranged product **3**, the experiment that comprised boiling the reagents in toluen, in the presence of 40% Et_2_NH for 7 h was the best choice (Table 1, entry 8). It is noteworthy that, for a similar reaction performed in the presence of only 20% Et_2_NH for 5 h, the ratio of the components reversed, as the ratio of adduct **2** to rearranged product **3** was 88–12 (Table 1, entry 9). Products **2** and **3** were obtained from the most successful experiments (Table 1, entries 1 and 8, respectively) in a yield of 86% and 75%, respectively, by column chromatography. It is noted that product **2** is the tetraethyl ester of etidronic acid, which is a first-generation dronic acid. Similar rearrangements were also observed during the base-promoted transformation of β-hydroxyphosphine oxides [18].

It was a challenge for us to prepare the hydroxymethylene bisphosphonates with mixed ester functions. Therefore, diethyl α-oxoethylphosphonate (**1**) was reacted with dimethyl phosphite in diethyl ether at 0 °C for 8 h in the presence of 5 and 20% Et_2_NH. To our surprise, the outcome of these two experiments was quite different: while in the first case, the expected adduct **4** was the major component (94%) (Table 2, entry 1), in the second case, the rearranged products (**5-1** and **5-2**) predominated (71% and 17%), (Table 2, entry 2). The Pudovik reaction was much less selective with dimethyl phosphite than with the diethyl counterpart (compare entry 2 of Table 2 with entry 2 of Table 1). The application of 5% Bu_2_NH led also to a selective addition, as adduct **4** was exclusively formed (Table 2, entry 3). The use of 40% Et_2_NH in diethyl ether at 0 °C for 8 h led to the predominant formation of the rearranged products **5-1** (76%) and **5-2** (21%) all together 97% (Table 2, entry 4). At the same time, the application of 5% DBA without any solvent at 120 °C for 20 min afforded the adduct **4** in a selectivity of 79% (Table 2, entry 5). To promote the rearrangement, the reaction of α-oxophosphonate (**1**) with dimethyl phosphite was performed in boiling toluene in the presence of 20% Et_2_NH for 5 h. Indeed, the rearranged products (**5-1** and **5-2**) predominated at 85% (Table 2, entry 6).

One can see that the nature of the amine (DEA or DBA) and its quantity (5–40%) have a major impact on the outcome of the reaction of diethyl α-oxoethylphosphonate (**1**) and dialkyl phosphite, while the temperature, as well as the use or lack of solvent, has a lesser effect. Adduct **4** and the mixture of rearranged products (**5-1** and **5-2**) were prepared from the best experiments (Table 2, entries 3 and 4) by column chromatography in yields of 87% and 76%, respectively.

Products **4**, **5-1**, and **5-2** exhibited ^31^P-NMR spectra comprising doublet patterns for each signal (see below and Experimental).

**Scheme 1:**
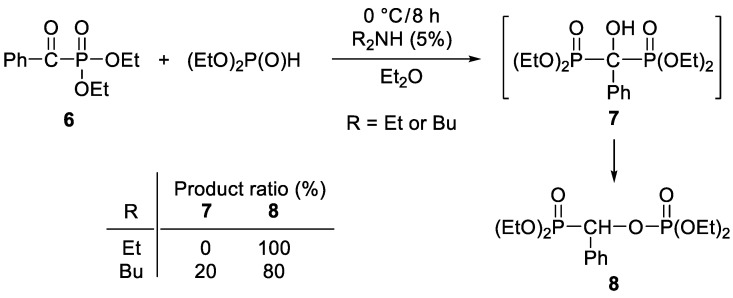
The reaction of diethyl α-oxobenzylphosphonate (**6**) with diethyl phosphite.

Then, diethyl oxobenzylphosphonate (**6**) was reacted with diethyl phosphite in the presence of 5% Et_2_NH and 5% Bu_2_NH in diethyl ether at 0 °C for 8 h. In the first case, exclusively the diethyl phosphonobenzylphosphate (**8**) formed by rearrangement of the Pudovik adduct (**7**) was present in the mixture (Scheme 1). It is noteworthy that when using Bu_2_NH as the catalyst, a mixture of 20% hydroxy-bisphosphonate (**7**) and 80% phosphono-phosphate (**8**) was obtained. Interrupting the Bu_2_NH-enhanced reaction of oxophosphonate (**6**) with of diethyl phosphite after 3 h (at 0 °C), the ratio of the components was reversed as there was 95% of adduct **7** and 5% of phosphono-phosphate (**8**) in the mixture. Adduct **7** was identified as an unstable intermediate (δ_P_ (CDCl_3_) 17.6; [M + Na]^+^_found_ = 403.1049; C_15_H_26_O_7_P_2_Na requires 403.1051). Adduct **7** slowly (after 3 weeks) rearranged to phosphono-phosphate (**8**) upon resting at 26 °C. Product **8** was isolated by column chromatography from the selective Et_2_NH-catalyzed reaction in a yield of 74%.

**Scheme 2:**
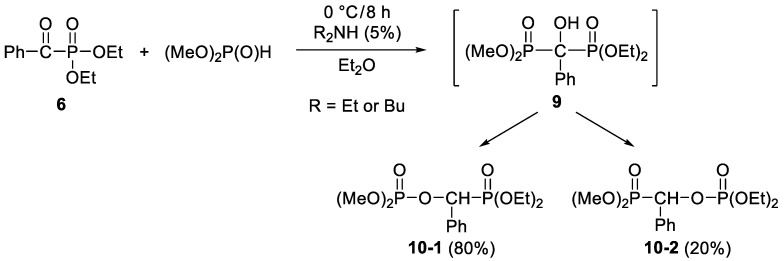
The reaction of diethyl α-oxobenzylphosphonate (**6**) with dimethyl phosphite.

Complete rearrangement followed the Pudovik reaction of oxobenzylphosphonate (**6**) with dimethyl phosphite using 5% of the secondary amine catalyst in diethyl ether solution at 0 °C (8 h) (Scheme 2). Due to the asymmetry of the intermediate **9**, the dialkyl phosphonobenzyl phosphate was formed as two isomers (**10-1** and **10-2**). Using Et_2_NH the yield was 81%, while the isomeric ratio was 80–20%.

The assignment of **5-1** and **10-1**, as well as **5-2** and **10-2** as the major and minor isomers, respectively, is tentative. However, considering the electrophilicity of the P atom in the (MeO)_2_P(O) and the (EtO)P(O) groups, the former is assumed to be more capable of being attacked by the hydroxy group, as the MeO substituent is somewhat less electron-donating than the EtO one. At the same time, steric effects may also play a role in the observed selectivity. The adduct with a C–Ph substituent (**9**) has an increased inclination for the rearrangement than the adduct with a C–Me unit (**4**) due to electronic effects.

**Scheme 3:**
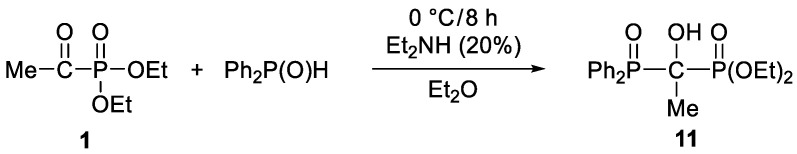
The reaction of diethyl α-oxoethylphosphonate (**1**) with diphenylphosphine oxide.

**Scheme 4:**
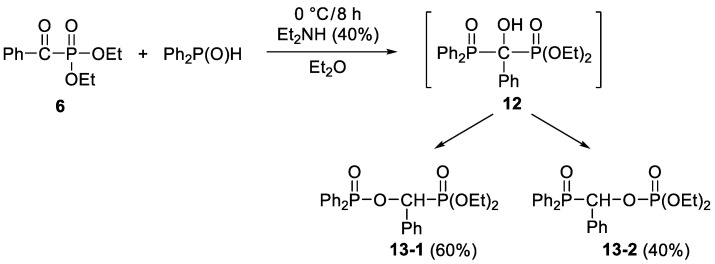
The reaction of diethyl α-oxobenzylphosphonate (**6**) with diphenylphosphine oxide.

In the final part of our experimental work, α-oxophosphonates **1** and **6** were reacted with diphenylphosphine oxide. While the interaction of α-oxoethylphosphonate (**1**) with diphenylphosphine oxide at 0 °C in the presence of 20% diethylamine in diethyl ether afforded the Pudovik adduct **11** (Scheme 3). The similar reaction of oxobenzylphosphonate (**6**) led to a 1:1 mixture of the two possible rearranged products; phosphinoyloxybenzylphosphonate (**13-1**), and phosphinoylbenzylphosphate (**13-2**) (Scheme 4). The ratio of isomers **13-1** and **13-2** was in accordance with the electrophilicity of the Ph_2_P(O) and (EtO)_2_P(O) moieties during rearrangement. However, due to the more sterically hindered Ph_2_P(O) moiety, the ratio of the isomers is more balanced (40–60%) compared to that of **10-1** and **10-2** (20–80%), due to the way that formation of a P–O bond occurs with the liberation of 385 kJ mol^–1^ [19].

Again, the adduct containing a phenyl group on the central carbon atom (as in **12**) seemed to be less stable compared to the methyl analogue **11**.

Adducts **2**, **4**, and **11**, as well as rearranged products **3**, **5**, **8**, **10**, and **13** were characterized by ^31^P, ^13^C and ^1^H-NMR, as well as with HRMS data. With the exception of compounds **2**, **3** and **4**, all other species are new derivatives.

The ^31^P-NMR spectral data were in accord with the different types of compounds synthesized. The symmetrical tetraethyl Pudovik adducts **2** and **7** revealed a signal at δ_P_ 20.3 and 17.6, respectively, while the diethyl–dimethyl derivative **4** appeared at δ_P_ 20.0 and 22.8 with a ^2^*J*_PP_ of 35.0 Hz. The adduct with (EtO)_2_P(O) and Ph_2_P(O) functions (**11**) had signals at δ_P_ 21.8 and 29.1 with a ^2^*J*_PP_ of 22.4 Hz.

The tetraethyl rearranged products **3** and **8** showed signals for phosphate and phosphonate moieties at δ_P_ −1.2/−1.1 and 20.3/16.7, respectively, with ^3^*J*_PP_ couplings of 32.0 and 34.9 Hz, respectively. The major isomers (**5-1** and **10-1**) of the diethyl–dimethyl derivatives revealed signals for the (EtO)_2_P(O)O and (MeO)_2_P(O)C units at δ_P_ 1.1/1.3 and 20.1/16.6, respectively, with ^3^*J*_PP_ couplings of 31.2 and 34.0 Hz, respectively. The major isomer (**13-1**) of the (EtO)_2_P(O)–Ph_2_P(O) derivative appeared at δ_P_ 17.2 and 34.7 (^3^*J*_PP_ = 26.7 Hz).

## 3. Experimental

### 3.1. General

The ^31^P, ^13^C, ^1^H-NMR spectra were taken on a Bruker DRX-500 spectrometer operating at 202.4, 125.7, and 500 MHz, respectively. The couplings are given in Hz. LC–MS measurements were performed with an Agilent 1200 liquid chromatography system, coupled with a 6130 quadrupole mass spectrometer equipped with an ESI ion source (Agilent Technologies, Palo Alto, CA, USA). High-resolution mass spectrometric measurements were performed using a Thermo Velos Pro Orbitrap Elite hybrid mass spectrometer in positive electrospray mode.

For the ^31^P, ^13^C and ^1^H-NMR spectra of the compounds prepared see Supplementary Materials.

### 3.2. Preparation of the α-Oxoethylphosphonates (***1*** and ***6***)

To 0.05 mol of the acid chloride (A: acetyl chloride, 3.6 mL; B: benzoyl chloride, 5.8 mL), 0.05 mol (8.6 mL) of triethyl phosphite was added dropwise at 0 °C with intensive stirring. Then, the mixture was allowed to warm to 26 °C and the stirring was continued for 3 h. In the case of “A”, α-oxoethylphosphonate (**1**) was obtained by distillation in vacuo. Yield: 4.9 g (55%), bp 130–132 °C/40 torr; bp [14] 86–88 °C/6 torr; δ_P_ (CDCl_3_) −2.8, δ_P_ [14] (CDCl_3_) 2.9. In the case of “B”, α-oxo(phenylmethyl)phosphonate (**6**) was prepared by column chromatography (using silica gel and 3% MeOH in DCM as the eluent). Yield: 8.9 g (83%); δ_P_ (CDCl_3_) 0.0, δ_P_ [14] (CDCl_3_) −0.85.

### 3.3. Synthesis of the Target Compounds

#### 3.3.1. Tetraethyl α-Hydroxy-ethylidenebisphosphonate (**2**)

(Table 1, Entry 4): Two point two millimoles (0.30 mL) of diethyl phosphite was added slowly to a mixture of 2.2 mmol (0.40 g) of diethyl α-oxoethylphosphonate (**1**) and 0.11 mmol (0.019 mL) of dibutylamine in diethyl ether (13 mL) at 0 °C whilst stirring. After an 8 h reaction time, the solvent was evaporated, and the crude product was obtained via purification with column chromatography (using DCM–MeOH 97:3 as the eluent on silica gel) to afford 0.57 g (82%) of adduct **2**. ^31^P-NMR (CDCl_3_) δ 20.3, δ_P_ [10] 20.8; ^13^C-NMR (CDCl_3_) δ 16.5 (t, *J* = 3.1 Hz, CH_2_CH_3_), 20.0 (t, *J* = 2.2 Hz, CCH_3_), 63.60–63.69 (m, CH_2_CH_3_), 71.43 (t, *J* = 155.2 Hz, CCH_3_); ^1^H-NMR (CDCl_3_) δ 1.35 (t, *J* = 7.1 Hz, 12H, CH_3_CH_2_), 1.64 (t, *J* = 16.1 Hz, 3H, CCH_3_), 4.20–4.27 (m, 8H, CH_2_O); [M + Na]^+^_found_ = 341.0891, C_10_H_24_O_7_P_2_Na requires 341.0895. 

#### 3.3.2. Diethyl 1-(Diethylphosphonoylethyl)phosphate (**3**) 

(Table 1, Entry 8): The mixture of 2.2 mmol (0.30 mL) of diethyl phosphite, 0.88 mmol (0.10 mL) of diethylamine and 2.2 mmol (0.40 g) of diethyl α-oxoethylphosphonate (**1**) in toluene (13 mL) was stirred at its boiling point for 7 h. Then, the solvent was evaporated, and the residue was obtained via purification using column chromatography (as described above) to give 0.53 g (75%) of the rearranged product **3**. ^31^P-NMR (CDCl_3_) δ_1_ −1.2 and δ_2_ 20.3 (^3^*J*_PP_ = 32.0 Hz); ^13^C-NMR (CDCl_3_) δ 16.0 (d, ^3^*J*_PC_ = 7.1 Hz, OCH_2_CH_3_), 16.4 and 16.5 (d, ^3^*J*_PC_ = 5.7 Hz, OCH_2_CH_3_), 16.6 (d, ^2^*J*_PC_ = 1.5 Hz, CCH_3_), 62.9 and 63.0 (d, ^2^*J*_PC_ = 6.8 Hz, OCH_2_), 64.0 and 64.1 (d, ^2^*J*_PC_ = 6.0 Hz, OCH_2_), 69.2 (dd, ^1^*J*_PC_ = 174.3 Hz, ^2^*J*_PC_ = 7.1 Hz, CCH_3_); ^1^H-NMR (CDCl_3_) δ 1.30–1.37 (m, 12H, CH_3_CH_2_), 1.57 (dd, *J*_1_ = 16.6 Hz, *J*_2_ = 7.0 Hz, 3H, CHCH_3_), 4.10–4.24 (m, 8H, CH_2_O), 4.67–4.72 (m, 1H, CHCH_3_); [M + Na]^+^_found_ = 341.0896, C_10_H_24_O_7_P_2_Na requires 341.0895. 

#### 3.3.3. Diethyl-Dimethyl α-Hydroxy-ethylidenebisphosphonate (**4**) 

(Table 2, Entry 3): Compound (**4**) was prepared as the tetraethyl counterpart **2** using 2.2 mmol (0.20 mL) of dimethyl phosphite instead of diethyl phosphite. Yield after chromatography: 0.57 g (87%). ^31^P-NMR (CDCl_3_) δ_1_ 20.0 and δ_2_ 22.8 (^2^*J*_PP_ = 35.0 Hz); ^13^C-NMR (CDCl_3_) δ 16.4 (d, *J* = 5.6 Hz, CH_3_CH_2_), 20.4 (bs, CCH_3_), 54.2 (dd, *J*_1_ = 9.7 Hz, *J*_2_ = 7.1 Hz, CH_3_O), 63.8 (dd, *J*_1_ = 7.2 Hz, *J*_2_ = 4.9 Hz, CH_2_O), 71.6 (t, *J* = 156.4 Hz, CP_2_); ^1^H-NMR (CDCl_3_) δ 1.37 (t, *J* = 7.0 Hz, CH_2_CH_3_, 6H), 1.68 (t, *J* = 16.1 Hz, CCH_3_, 3H), 3.88 (d, *J* = 10.5 Hz, OCH_3_, 6H), 4.21–4.32 (m, OCH_2_, 4H); [M + Na]^+^_found_ = 313.0574, C_8_H_20_O_7_P_2_Na requires 313.0582. 

#### 3.3.4. Dimethyl 1-(Diethylphosphonoylethyl)phosphate (**5-1**) and Diethyl 1-(Dimethylphosphonoylethyl)phosphate (**5-2**) 

(Table 2, entry 4): Compounds (**5-1**) and (**5-2**) were prepared via the reaction of 2.2 mmol (0.40 g) of diethyl α-oxoethylphosphonate (**1**) with 2.2 mmol (0.20 mL) of dimethyl phosphite in the presence of 0.88 mmol (0.10 mL) of diethylamine in diethyl ether (13 mL) at 0 °C for 8 h. The work-up including chromatography was performed as described above to furnish 0.49 g (76%) of the 76:21 mixture of **5-1** and **5-2**. ^31^P-NMR (CDCl_3_) major: δ_1_ 1.1 and δ_2_ 20.1 (^3^J_PP_ = 31.2 Hz); minor: δ_1_ −1.2 and δ_2_ 22.7 (^3^*J*_PP_ = 29.7 Hz). ^13^C-NMR (CDCl_3_) major: δ 16.38 and 16.43 (d, ^3^*J*_PC_ = 5.5, OCH_2_CH_3_), 16.6 (CCCH_3_), 54.4 and 54.5 (^2^*J*_PC_ = 6.2 Hz, OCH_3_), 63.0 and 63.1 (d, ^2^*J*_PC_ = 6.5 Hz, OCH_2_), 69.4 (dd, ^1^*J*_PC_ = 174.5 Hz, ^2^*J*_PC_ = 6.8 Hz, CCH_3_); minor: δ 16.0 (d, ^3^*J*_PC_ = 6.8 Hz, OCH_2_CH_3_), 16.6 (CCH_3_), 53.4 and 53.6 (d, ^2^*J*_PC_ = 6.5 Hz, OCH_3_), 64.1 and 64.2 (d, ^2^*J*_PC_ = 6.1 Hz, OCH_2_), 68.8 (dd, ^1^*J*_PC_ = 174.1 Hz, ^1^*J*_PC_ = 7.0 Hz, CCH_3_). ^1^H-NMR (CDCl_3_) major: δ 1.32 (t, *J* = 7.0 Hz, CH_2_CH_3_, 6H), 1.54 (dd, *J* = 16.7 Hz, *J* = 7.0 Hz, CCH_3_, 3H), 3.75 and 3.77 (d, *J* = 11.5 Hz, OCH_3_, 6H), 4.13–4.20 (m, OCH_2_, 4H); [M + Na]^+^_found_ = 313.0581, C_8_H_20_O_7_P_2_Na requires 313.0582.

#### 3.3.5. Diethyl (Diethylphosphonoylbenzyl)phosphate (**8**)

A total of 1.7 mmol (0.20 mL) of diethyl phosphite was added to a mixture of 1.7 mmol (0.40 g) of diethyl α-oxobenzylphosphonate (**6**) and 0.085 mmol (0.008 mL) of diethylamine in diethyl ether (13 mL) at 0 °C whilst being stirred. After an 8 h reaction time, the solvent was evaporated, and the crude product was obtained by purification via column chromatography (as described above) to furnish 0.48 g (74%) of phosphonoyl-phosphate (**8**). ^31^P-NMR (CDCl_3_) δ_1_ −1.1 and δ_2_ 16.7 (^3^*J*_PP_ = 34.9 Hz); ^13^C-NMR (CDCl_3_) δ 15.8 and 15.9 (d, ^3^*J*_PC_ = 7.2 Hz, OCH_2_CH_3_), 16.3 and 16.4 (d, ^3^*J*_PC_ = 5.8 Hz, OCH_2_CH_3_), 63.4 and 63.5 (d, ^2^*J*_PC_ = 6.8 Hz, OCH_2_), 63.9 and 64.1 (d, ^2^*J*_PC_ = 5.8 Hz, OCH_2_), 75.2 (dd, ^1^*J*_PC_ = 172.2 Hz, ^2^*J*_PC_ = 7.1 Hz, CPh), 128.0 (^3^*J*_PC_ = 6.0 Hz, C_β_), 128.4 (^4^*J*_PC_ = 2.1 Hz, C_γ_), 129.0 (C_δ_), 133.7 (C_α_); ^1^H-NMR (CDCl_3_) δ 1.12 (t, *J* = 7.1 Hz, 3H, OCH_2_CH_3_), 1.23 (t, *J* = 7.1 Hz, 3H, OCH_2_CH_3_), 1.27 (t, *J* = 7.0 Hz, 3H, OCH_2_CH_3_), 1.29 (t, *J* = 7.1 Hz, 3H, OCH_2_CH_3_), 3.82–4.19 (m, 8H, OCH_2_), 5.55 (dd, *J*_1_ = 13.6 Hz, *J*_2_ = 10.5 Hz, 1H, CH), 7.34–7.40 and 7.50–7.54 (m, 5H, Ph); [M + Na]^+^_found_ = 403.1057, C_15_H_26_O_7_P_2_Na requires 403.1051.

#### 3.3.6. Dimethyl (Diethylphosphonoylbenzyl)phosphate (**10-1**) and Dimthylphosphonoylbenzyl)phosphate (**10-2**)

Compounds (**10-1**) and (**10-2**) were prepared by the reaction of 0.83 mmol (0.20 g) of diethyl α-oxobenzylphosphonate (**6**) with 0.83 mmol (0.10 mL) of dimethyl phosphite in the presence of 0.042 mmoL (0.004 mL) of diethylamine in diethyl ether (13 mL) at 0 °C for 8 h. The work-up, including chromatography, was performed as described above to give 0.24 g (81%) of the 8:2 mixture of **10-1** and **10-2**. ^31^P-NMR (CDCl_3_) major: δ_1_ 1.3 and δ_2_ 16.6 (^3^*J*_PP_ = 34.0 Hz); minor δ_1_ −1.2 and δ_2_ 19.1 (^3^*J*_PP_ = 33.6 Hz). ^13^C-NMR (CDCl_3_) major: δ 16.4 and 16.5 (d, ^3^*J*_PC_ = 5.8 Hz, OCH_2_CH_3_), 54.4 and 54.7 (d, *J*_PC_ = 5.9 Hz, OCH_3_), 63.68 and 63.70 (d, *J*_PC_ = 6.8 Hz, OCH_2_), 75.1 (dd, ^1^*J*_PC_ = 172.4 Hz, ^2^*J*_PC_ = 6.9 Hz, CPh), 128.1 (^2^*J*_PC_ = 5.9 Hz, C_β_), 128.6 (^4^*J*_PC_ = 2.1 Hz, C_γ_), 129.27 (C_δ_), 133.6 (C_α_); minor: δ 15.9 and 16.1 (d, ^3^*J*_PC_ = 7.2 Hz, OCH_2_CH_3_), 54.1 and 54.2 (d, *J*_PC_ = 6.9 Hz, OCH_3_), 64.2 and 64.4 (d, *J*_PC_ = 5.8 Hz, OCH_2_), 74.5 (dd, ^1^*J*_PC_ = 172.3 Hz, ^2^*J*_PC_ = 6.8 Hz, CPh), 128.1 (^2^*J*_PC_ = 5.9 Hz, C_β_), 128.7 (^4^*J*_PC_ = 2.0 Hz, C_γ_), 129.32 (C_δ_), 133.5 (C_α_). ^1^H-NMR (CDCl_3_) δ 1.22 and 1.30 (t, ^3^*J*_HH_ = 7.1 Hz) for the major isomer, and δ 1.10 and 1.27 (t, ^3^*J*_HH_ = 7.1 Hz) for the minor isomer (total intensity: 6H, OCH_2_CH_3_); 3.54 and 3.75 (d, *J*_PH_ = 11.4 Hz) for the major isomer, and 3.68 and 3.77 (d, ^3^*J*_PH_ = 10.7 Hz) for the minor isomer (total intensity: 4H, OCH_3_); 3.84–4.18 (m, 4H, OCH_2_), 5.56 (dd, *J*_PH_ = 13.4 Hz, *J*_PH_ = 10.5 Hz) for the major isomer, and 5.60 (dd, *J*_1_ = 13.4 Hz, *J*_2_ = 10.5 Hz) for the minor isomer (total intensity: 1H, CH); 7.34–7.42 and 7.50–7.54 (m, 5H, Ar); [M + Na]^+^_found_ = 375.0735, C_13_H_22_O_7_P_2_Na requires 375.0738. 

#### 3.3.7. Diethyl 1-Diphenylphosphinoyl-1-hydroxy-ethylphosphonate (**11**) 

A total of 1.5 mmoL (0.27 g) of diethyl α-oxoethylphosphonate (**1**) in diethyl ether (3 mL) was added dropwise at 0 °C to a mixture of 1.5 mmol (0.30 g) of diphenylphosphine oxide and 0.30 mmol (0.03 mL) of diethylamine in diethyl ether (10 mL). Then, the mixture was stirred at 0 °C for 8 h. The precipitated material was removed by filtration, washed with diethyl ether, and the residue was recrystallized from acetone. Yield: 0.42 g (74%) of a white crystalline compound. mp: 170–171 °C. ^31^P-NMR (CDCl_3_) δ_1_ 21.8 and δ_2_ 29.1 (^2^*J*_PP_ = 22.4 Hz); ^13^C-NMR (CDCl_3_) δ 16.0 and 16.4 (d, ^3^*J*_PC_ = 5.7 Hz, OCH_2_CH_3_), 20.4 (s, CCH_3_), 63.4 and 63.6 (d, ^2^*J*_PC_ = 7.5 Hz, OCH_2_), 74.4 (dd, ^1^*J*_PC_ = 154.9 Hz, ^1^*J*_PC_ = 79.0 Hz, CCH_3_), 127.8 and 128.0 (d, ^3^*J*_PC_ = 11.7 Hz, C_γ_*), 131.4 (d, ^1^*J*_PC_ = 97.3 Hz, C_α_), 131.5 (s, C_δ_), 131.6 (d, ^4^*J*_PC_ = 3.0 Hz, C_δ_), 132.5 and 132.7 (d, ^2^*J*_PC_ = 8.6 Hz, C_β_*), * may be reversed; ^1^H-NMR (CDCl_3_) δ 1.08 and 1.25 (t, ^3^*J*_HH_ = 7.1 Hz, 6H, CH_2_CH_3_), 1.68 (t, ^3^*J*_P2H_ = 16.0 Hz, 3H, CCH_3_), 1.87 (s, 1H, OH), 3.74–4.26 (m, 4H, OCH_2_), 7.38–7.68 and 8.04–8.25 (m, 10H, ArH); [M + H]^+^_found_ = 383.1179, C_18_H_25_O_5_P_2_ requires 383.1177.

#### 3.3.8. Diethyl (Diphenylphosphinoyloxybenzyl)phosphonate and Diethyl (Diphenylphosphinoylbenzyl)phosphate (**13-1** and **13-2**)

This reaction was performed as described above, with the difference that 1.7 mmol (0.4 g) of diethyl oxobenzylphosphonate (**6**) was used instead of phosphonate **1** to afford 0.47 g (70%) of product **13**, comprising isomers **13-1** and **13-2** in a ratio of 6:4. **13-1**: ^31^P-NMR (CDCl_3_) δ_1_ 17.2 and δ_2_ 34.7 (^3^*J*_PP_ = 26.7 Hz) (60%); ^13^C-NMR (CDCl_3_) δ 16.2 and 16.3 (d, ^3^*J*_PC_ = 5.8 Hz, OCH_2_CH_3_), 63.3 and 63.5 (d, ^2^*J*_PC_ = 6.9 Hz, OCH_2_), 72.0 (dd, ^1^*J*_PC_ = 172.6 Hz, ^2^*J*_PC_ = 7.0 Hz, CPh), the aromatic range was rather complex between δ 128.0–132.6; ^1^H-NMR (CDCl_3_) δ 1.09 and 1.18 (t, *J* = 7.1 Hz, CH_2_CH_3_, 6H), 3.78–4.15 (m, OCH_2_, 4H), 5.63 (dd, *J*_1_ = 13.5 Hz, *J*_2_ = 11.2 Hz, CHPh, 1H), aromatic region: 7.15–7.98 (m). **13-2**: ^31^P-NMR (CDCl_3_) δ_1_ –1.5 and δ_2_ 28.6 (^3^*J*_PP_ = 31.3 Hz (40%); ^13^C-NMR (CDCl_3_) δ 15.6 and 15.8 (d, ^3^*J*_PC_ = 7.4 Hz, OCH_2_CH_3_), 63.8 and 63.9 (d, ^2^*J*_PC_ = 6.0 Hz, OCH_2_), 77.4 (dd, ^1^*J*_PC_ = 85.7 Hz, ^2^*J*_PC_ = 7.9 Hz, CPh), the aromatic range was rather complex between 128.0 and 132.6; ^1^H-NMR (CDCl_3_) δ 0.90 and 0.96 (t, *J* = 7.1 Hz, CH_2_CH_3_, 6H), 3.41–3.70 (m, OCH_2_, 4H), 6.06 (dd, *J*_1_ = 9.7 Hz, *J*_2_ = 4.4 Hz, CHPh, 1H), aromatic region: 7.15–7.98 (m); [M + Na]^+^_found_ = 467.1154, C_23_H_26_O_5_P_2_Na requires 467.1153.

## 4. Conclusions

In summary, the Pudovik reaction between α-oxophosphonates (ZC(O)P(O)(OEt)_2_, Z = Me or Ph) and Y_2_P(O)H reagents (Y = EtO, MeO, and Ph) may lead to the corresponding adducts (Y_2_P(O)C(OH)ZP(O)(OEt)_2_) and/or their rearranged versions. The outcome mostly depended on the Z substituent, the quantity of the dialkylamine (DAA) catalyst, and, to a lesser extent, on the nature of the DAA and Y substituents, as well as on the temperature and the solvent. In a few cases, time also had an influence on the course of the reaction. In cases where Z = Me, the adducts were the primary products, but with suitable modifications the reactions could be tuned to yield the rearranged derivatives. At the same time, in cases where Y = Ph, the corresponding adducts were only intermediates that were converted spontaneously to their rearranged versions. This phenomenon was explained by electronic factors. In reaction with dimethyl phosphite and diphenylphosphine oxide, the rearranged species comprised two isomers.

## Figures and Tables

**Table 1:** The reaction of diethyl α-oxoethylphosphonate (**1**) with diethyl phosphite under different conditions.

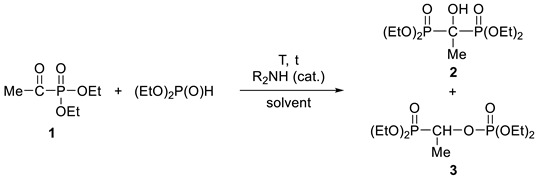							
Exp.	Catalyst (%)	Solvent	T (°C)	t	Product Composition (%) ^a^		Yield (%)
					2	3	
1	Et_2_NH (5)	Et_2_O	0	8 h	100	0	86% of **2**
2	Et_2_NH (20)	Et_2_O	0	8 h	98	2	
3	Et_2_NH (40)	Et_2_O	0	8 h	87	13	
4	Bu_2_NH (5)	Et_2_O	0	8 h	99	1	82% of **2**
5	Et_2_NH (40)	–	120	20 min ^b^	66	34	
6	Et_2_NH (40)	–	135	20 min	51	49	
7	Bu_2_NH (5)	–	120	20 min	94	6	
8	Et_2_NH (40)	PhMe	110	7 h ^c^	5	95	75% of **3**
9	Et_2_NH (20)	PhMe	110	5 h	88	12	

^a^ On the basis of relative ^31^P-NMR intensities; ^b^ On further heating for 3 h at 135 °C, the ratio of **2** and **3** became 32–68%; ^c^ Extrapolated reaction time.

**Table 2:** The reaction of diethyl α-oxoethylphosphonate (**1**), with dimethyl phosphite under different conditions.

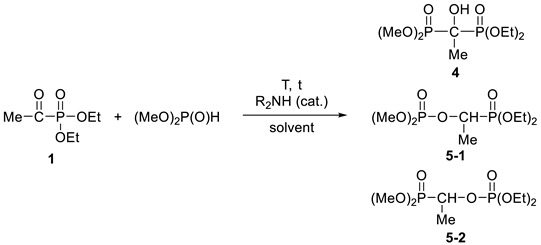								
Exp.	Catalyst (%)	Solvent	T (°C)	t	Product Composition (%) ^a^			Yield (%)
					4	5-1	5-2	
1	Et_2_NH (5)	Et_2_O	0	8 h	94	4	2	
2	Et_2_NH (20)	Et_2_O	0	8 h	12	71	17	
3	Bu_2_NH (5)	Et_2_O	0	8 h	100	0	0	87% (**4**)
4	Et_2_NH (40)	Et_2_O	0	8 h	3	76	21	76% (**5**)
5	Bu_2_NH (5)	–	120	20 min	79	17	4	
6	Et_2_NH (20)	PhMe	110	5 h	15	64	21	

^a^ On the basis of relative ^31^P-NMR intensities.

## Data Availability

Not applicable.

## Supplementary Materials

The following are available online. Copies of the ^31^P, ^13^C and ^1^H-NMR spectra of the compounds prepared.

## Sample Availability

Samples of the compounds are not available from the authors.
