# Supplementary material for: Tetraalkyl Hydroxymethylene-bisphosphonate and Dialkyl 1-Diphenylphosphinoyl-1-hydroxy-ethylphosphonate Derivatives by the Pudovik Reaction and Their Rearranged Products
Source: Molecules. 2021 Dec 14;26(24):7575. doi: 10.3390/molecules26247575 (PMC8707796; doi:10.3390/molecules26247575)
Supplement: Supplementary file 1 [file molecules-26-07575-s001.zip › molecules-1484067-supplementary.pdf]

molecules

MDPI

Supplementary Materials

Tetraalkyl Hydroxymethylene-bisphosphonate and Dialkyl 1-Diphenylphosphinoyl-1-hydroxy-ethylphosphonate Derivatives by the Pudovik Reaction and Their Rearranged Products

Zsuzsanna Szalai and György Keglevich *

Department of Organic Chemistry and Technology, Budapest University of Technology and Economics, 1521 Budapest, Hungary

^{31}P, ^{13}C and ^{1}H NMR spectra of products

2, **3**, **4**, **5-1** + **5-2**, **8**, **10-1** + **10-2**, **11** and **13-1** + **13-2**

Tetraethyl a-hydroxy-ethylidenebisphosphonate SzZs18+21/8.fr. 31P 300MHz

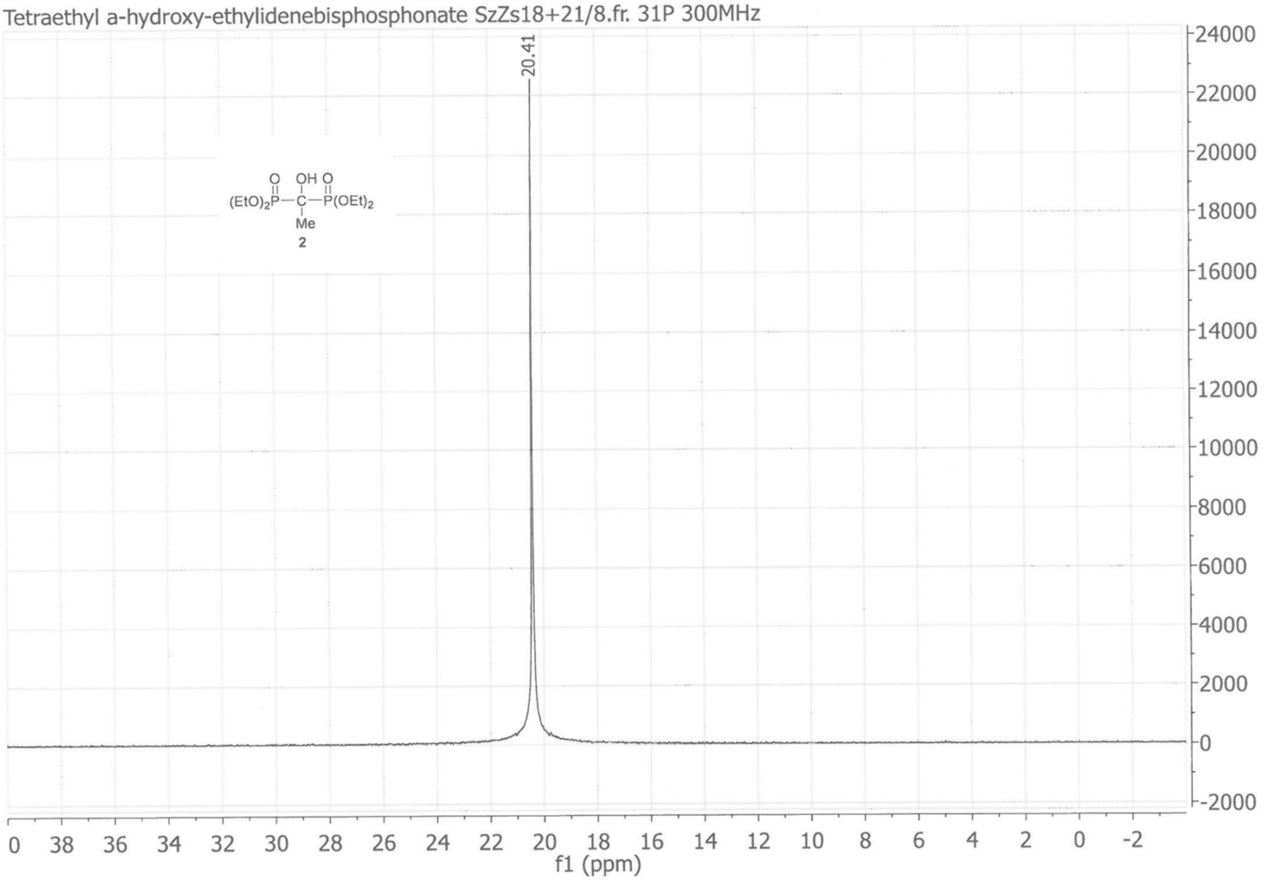

Tetraethyl a-hydroxy-ethylidenebisphosphonate 18+21/8.fr. 13C 300MHz

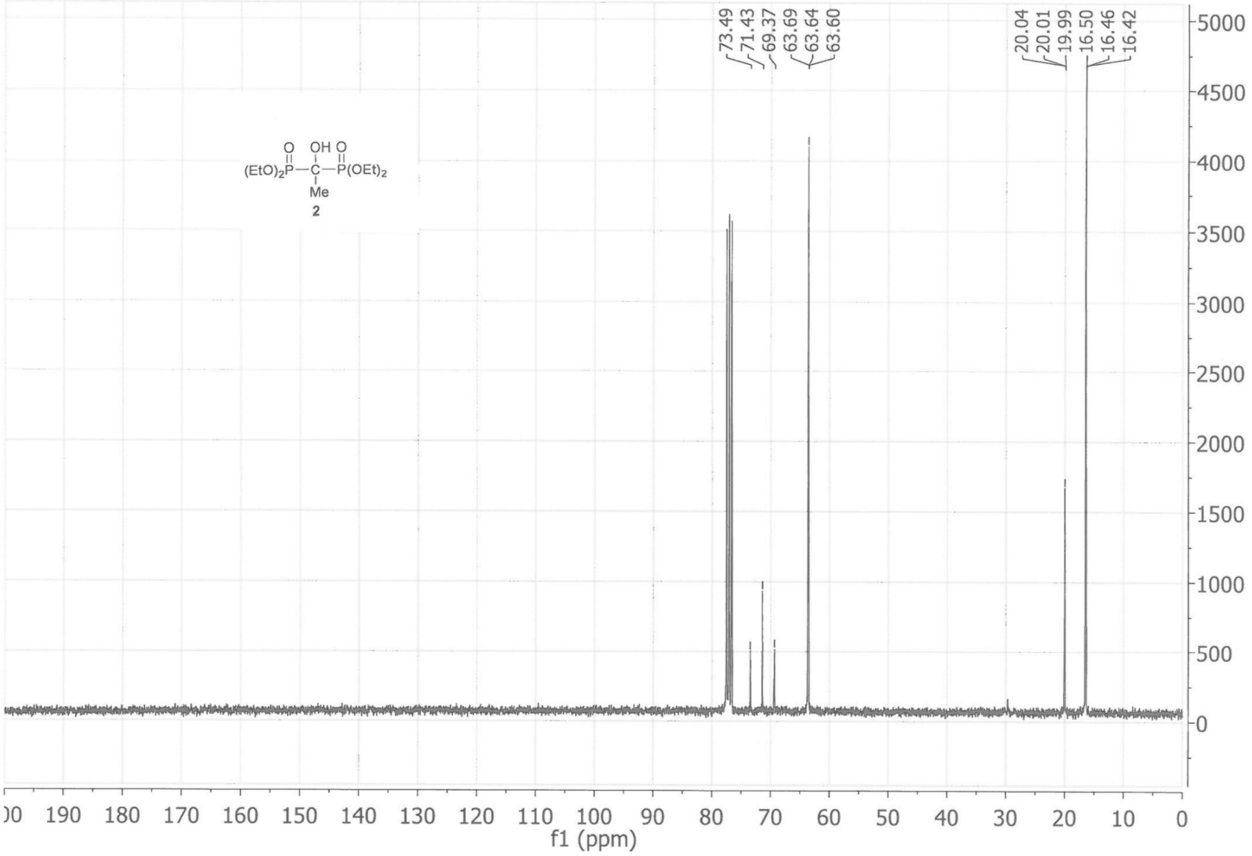

Tetraethyl a-hydroxy-ethylidenebisphosphonate 18+21/8.fr. 1H 500MHz

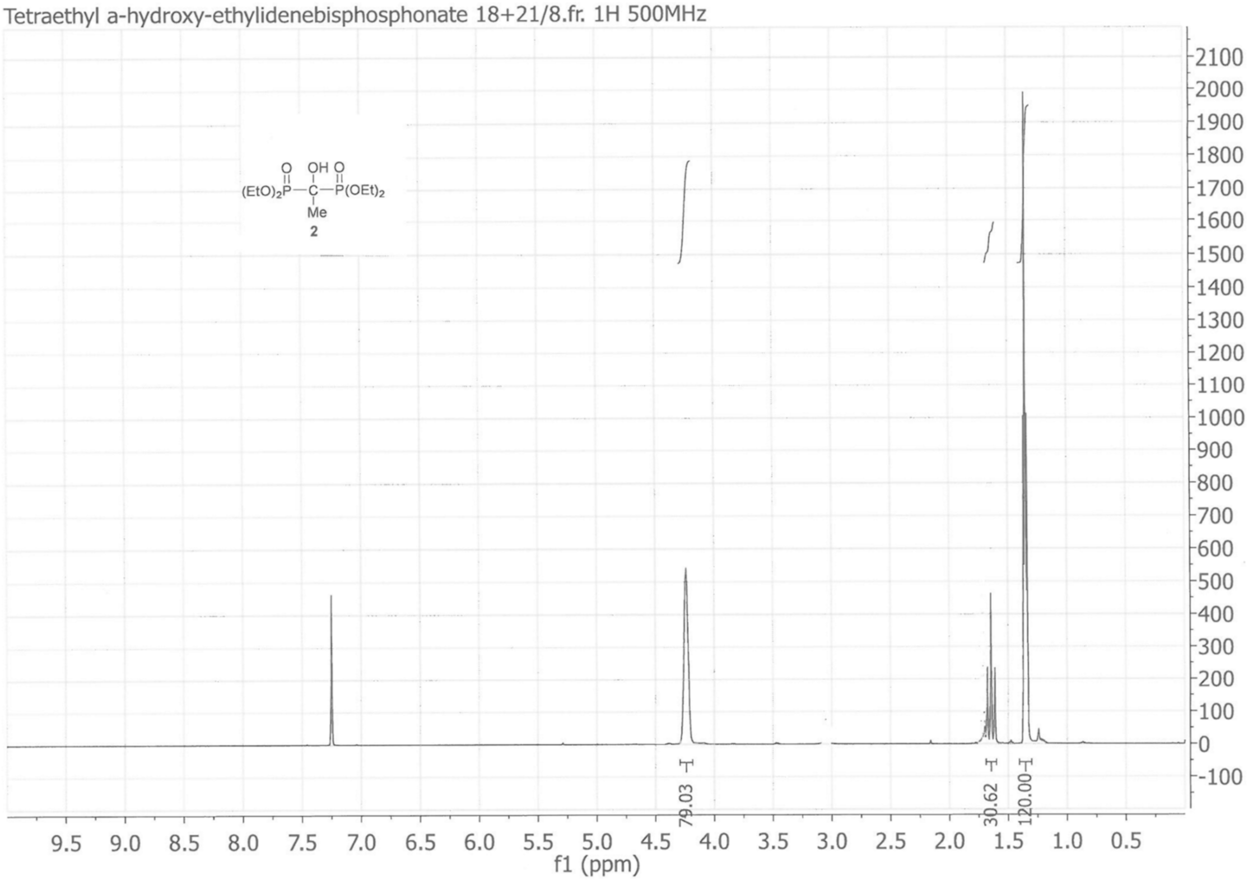

Diethyl 1-(diethylphosphonoylethyl)phosphate SzZs42/2.fr. 31P 500MHz

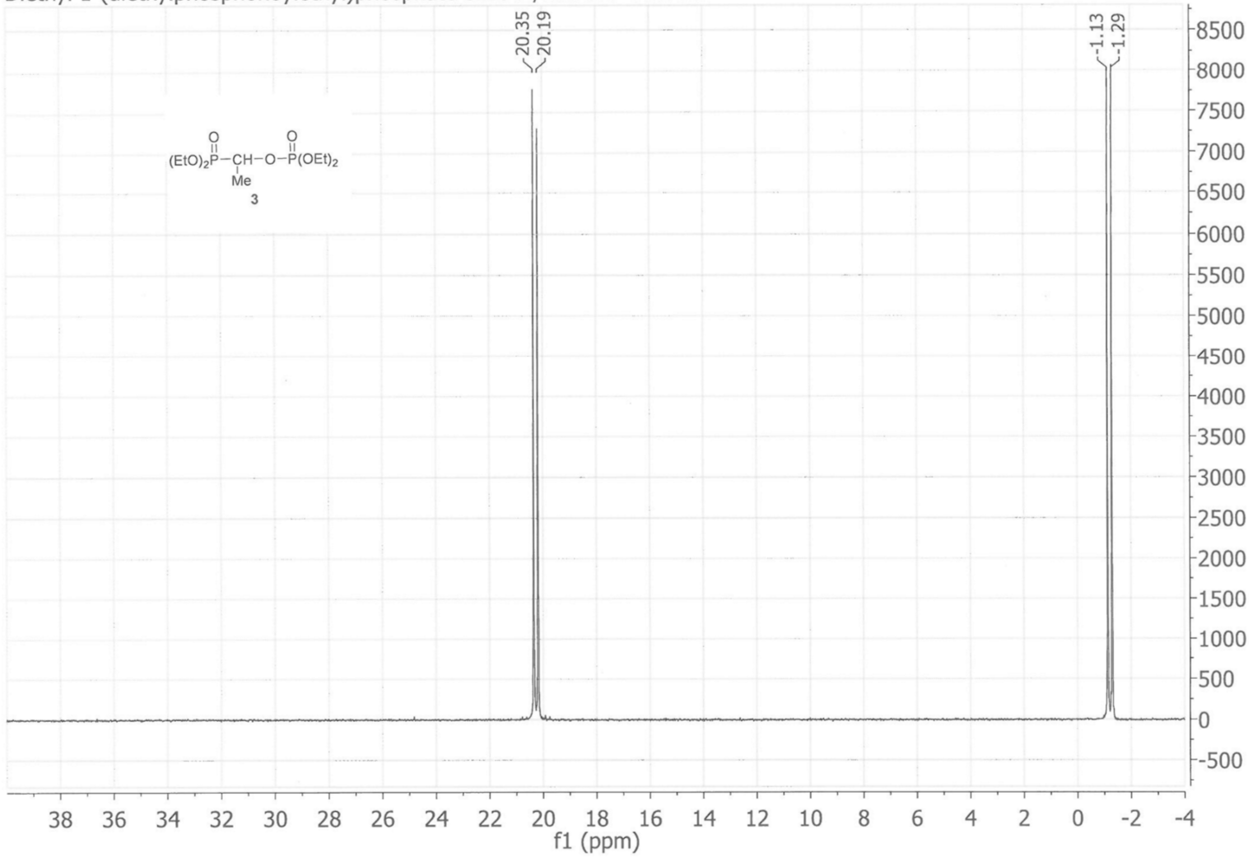

Diethyl 1-(diethylphosphonoylethyl)phosphate SzZs42/2.fr. 13C 300MHz

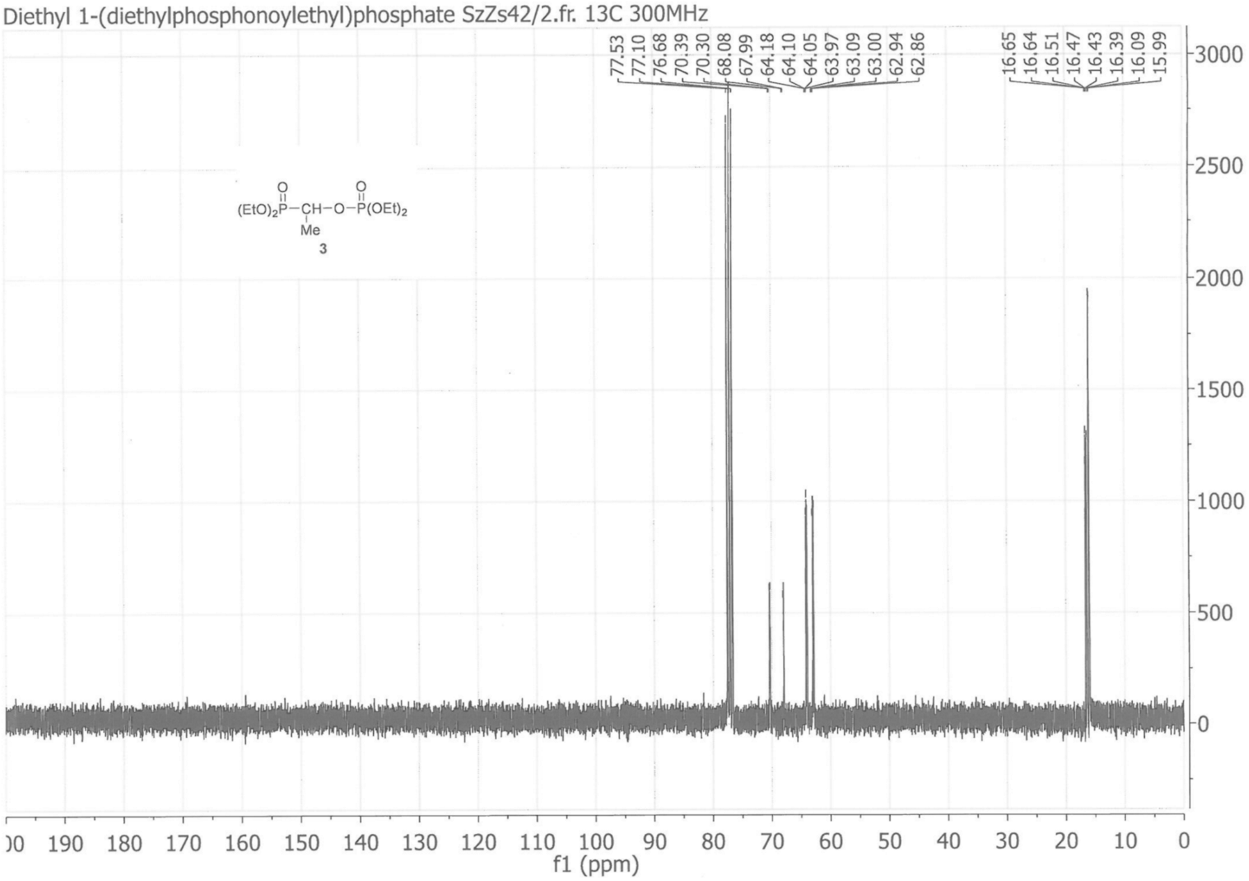

Diethyl 1-(diethylphosphonoylethyl)phosphate SzZs42/2.fr. 1H 500MHz

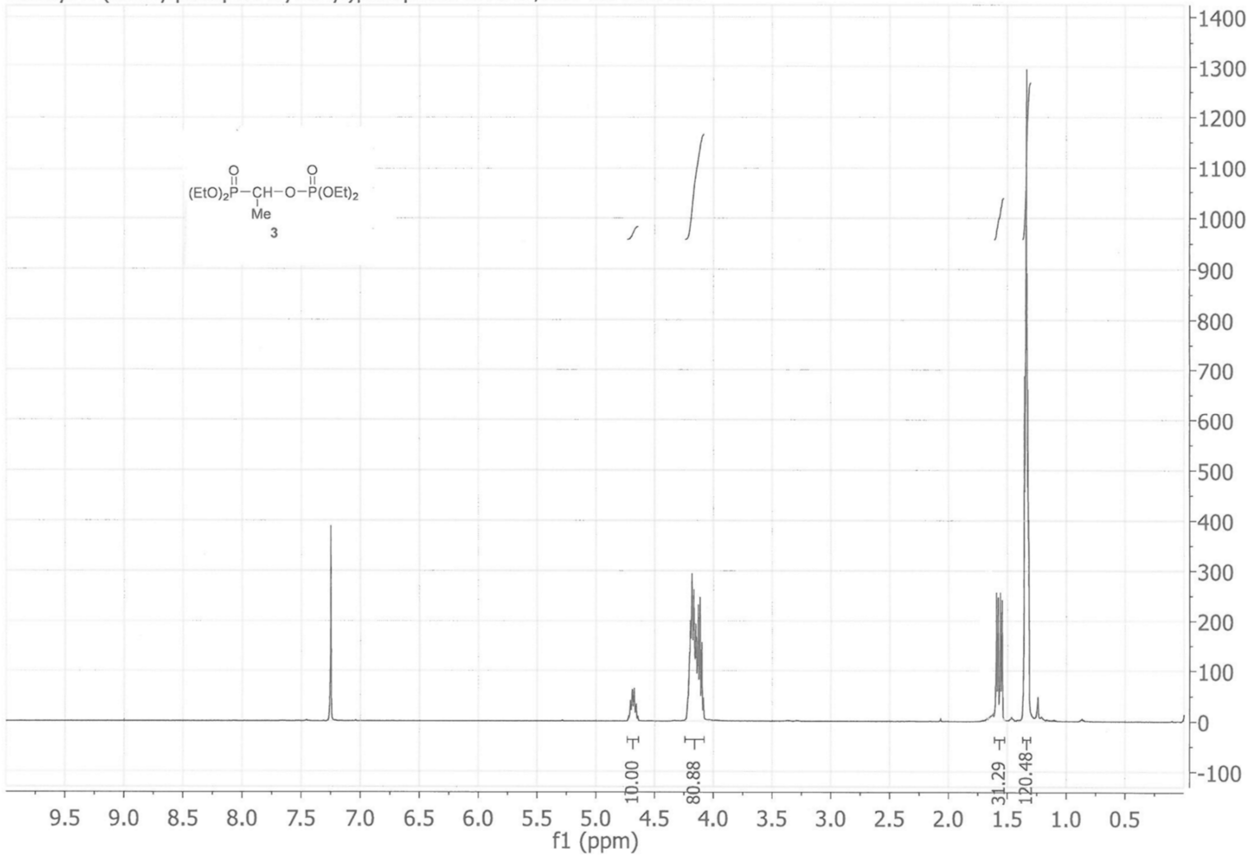

Diethyl dimethyl a-hydroxy-ethylidenebisphosphonate SzZs44ism/6.fr. 31P 500MHz

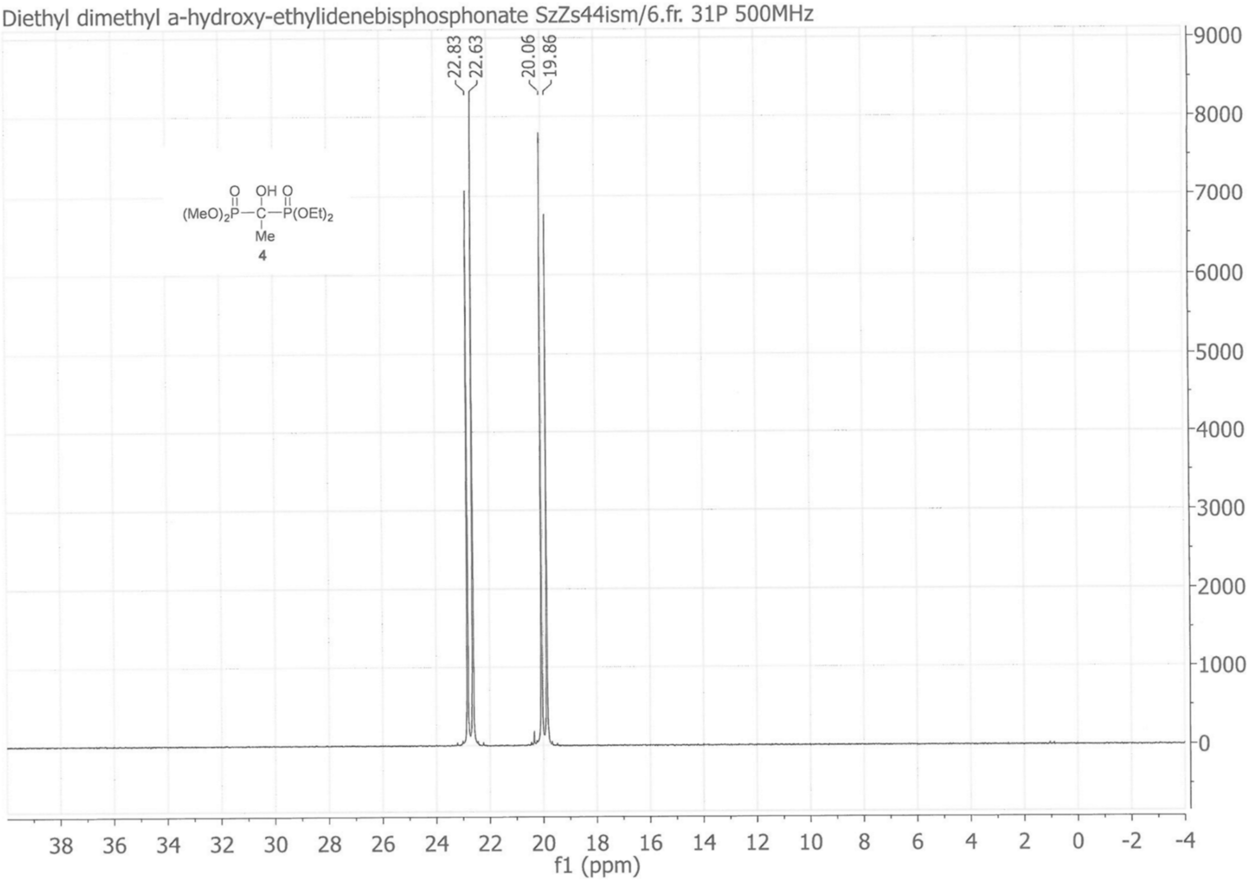

Diethyl dimethyl a-hydroxy-ethylidenebisphosphonate SzZs44ism/6.fr. 13C 300MHz

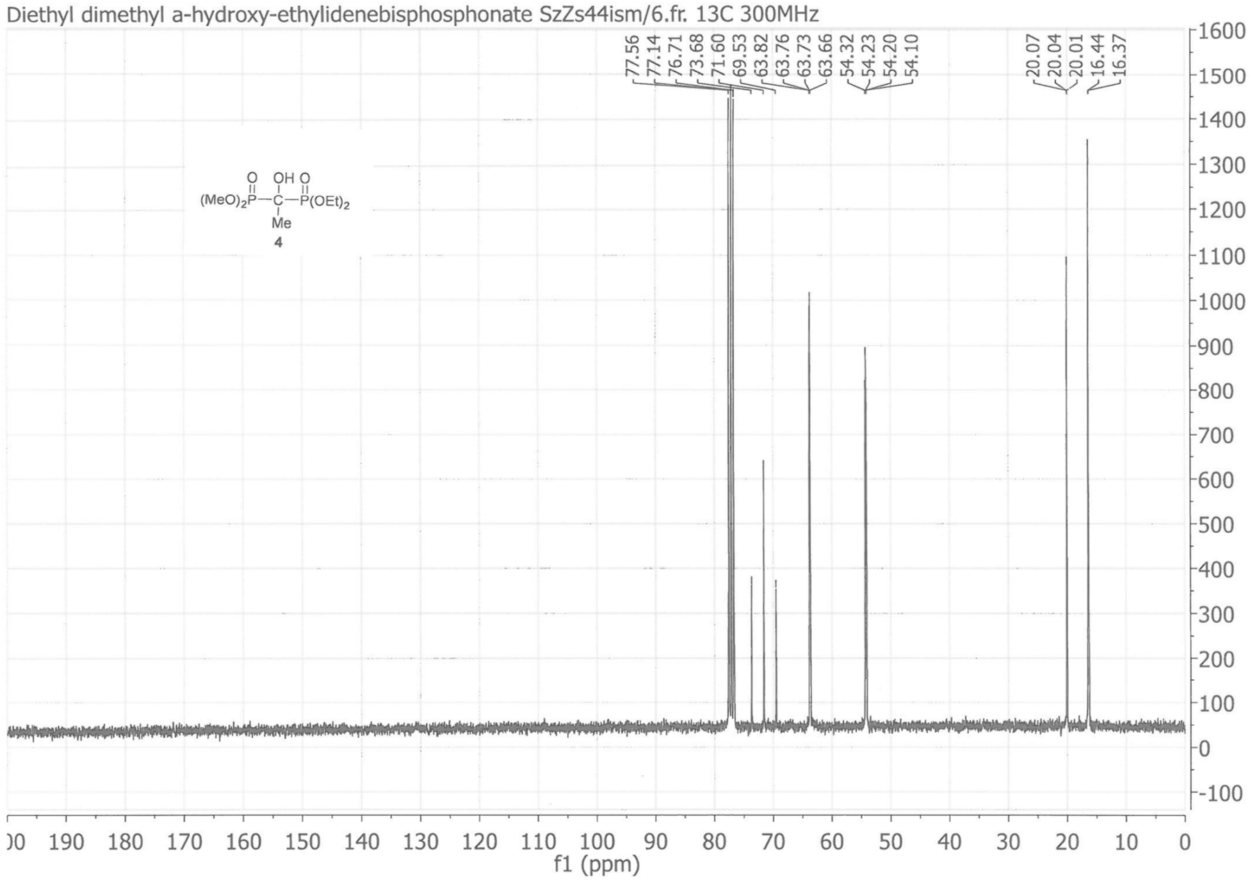

Diethyl dimethyl a-hydroxy-ethylidenebisphosphonate SzZs44ism/6.fr. 1H 300MHz

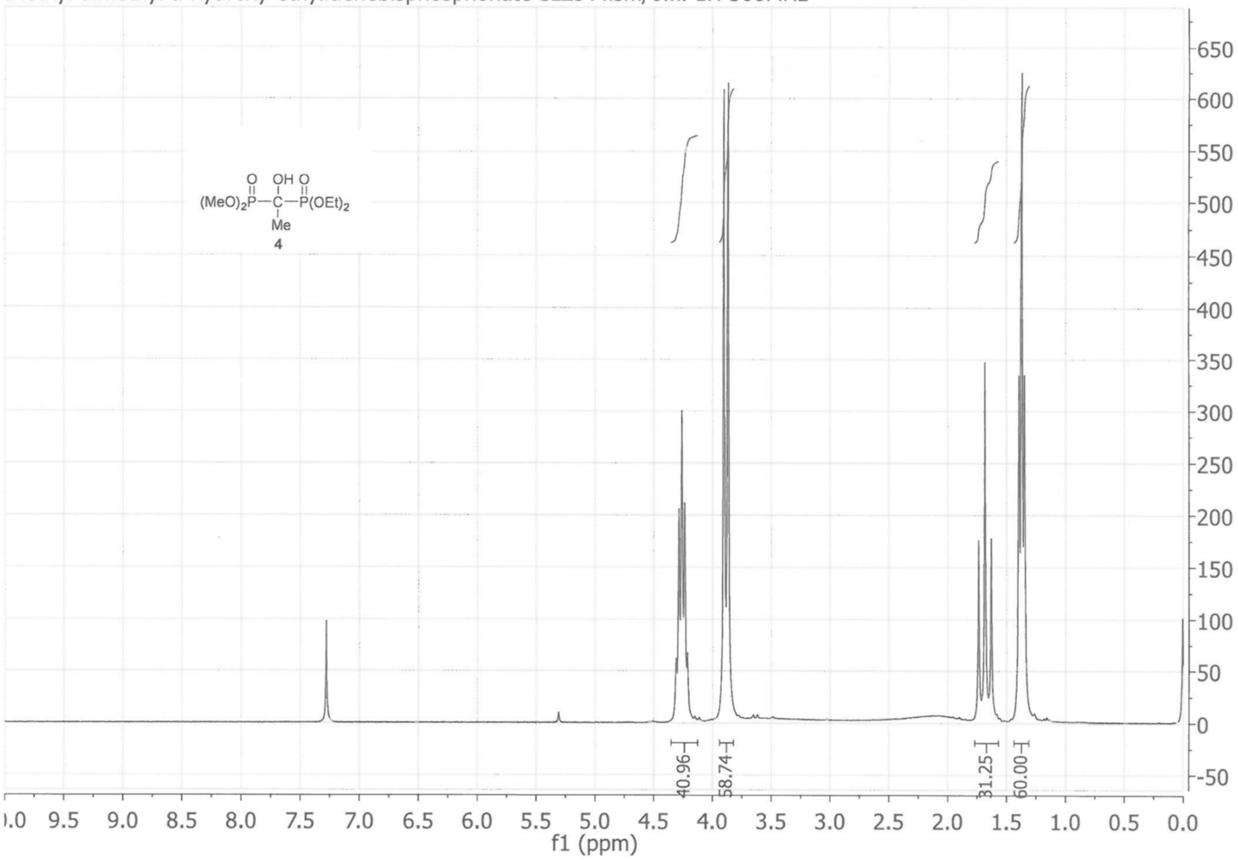

Diethyl 1-(dimethylphosphonoylethyl)phosphate and Dimethyl 1-(diethylphosphonoylethyl)phosphate SzZs43ism/2/3.fr. 31P 500M

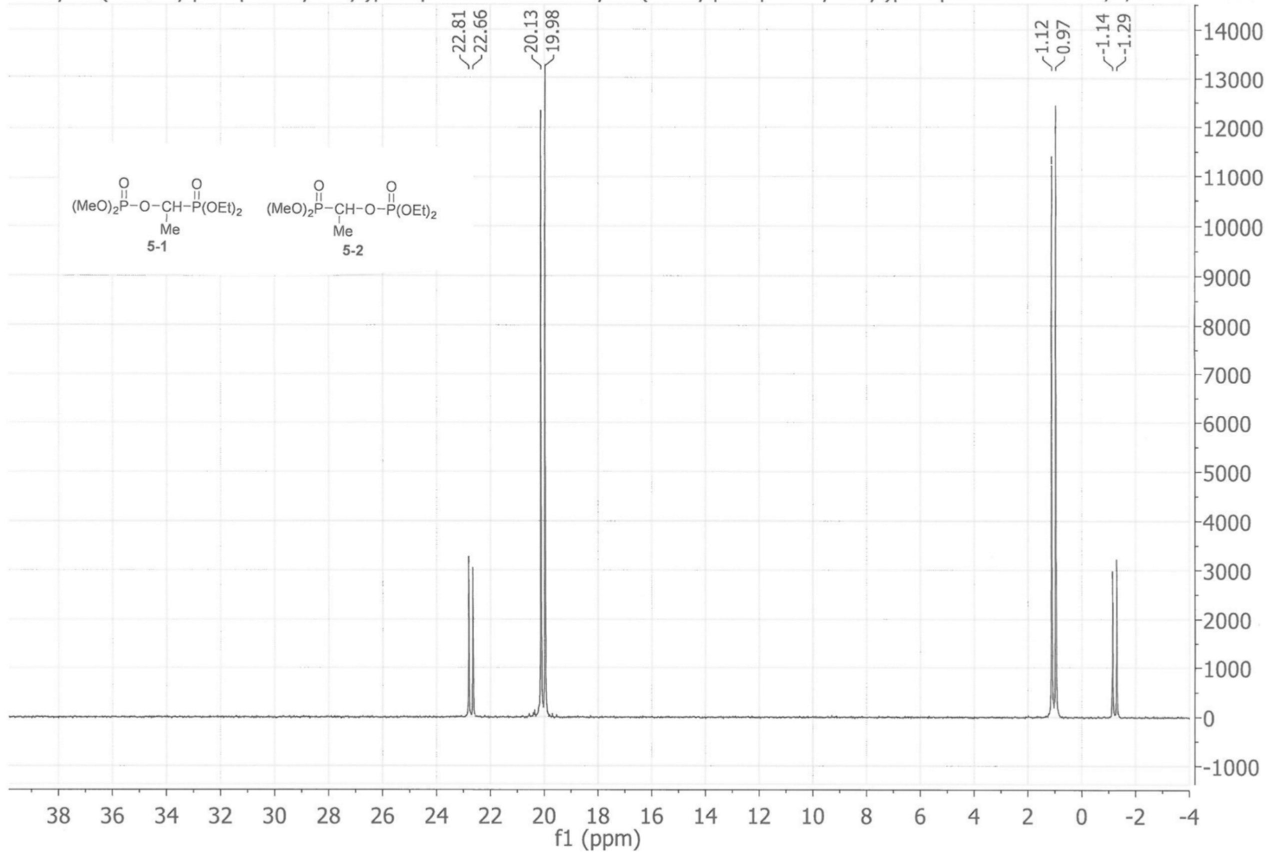

Diethyl 1-(dimethylphosphonoylethyl)phosphate and Dimethyl 1-(diethylphosphonoylethyl)phosphate SzZs43ism/2/3.fr. 13C 300M

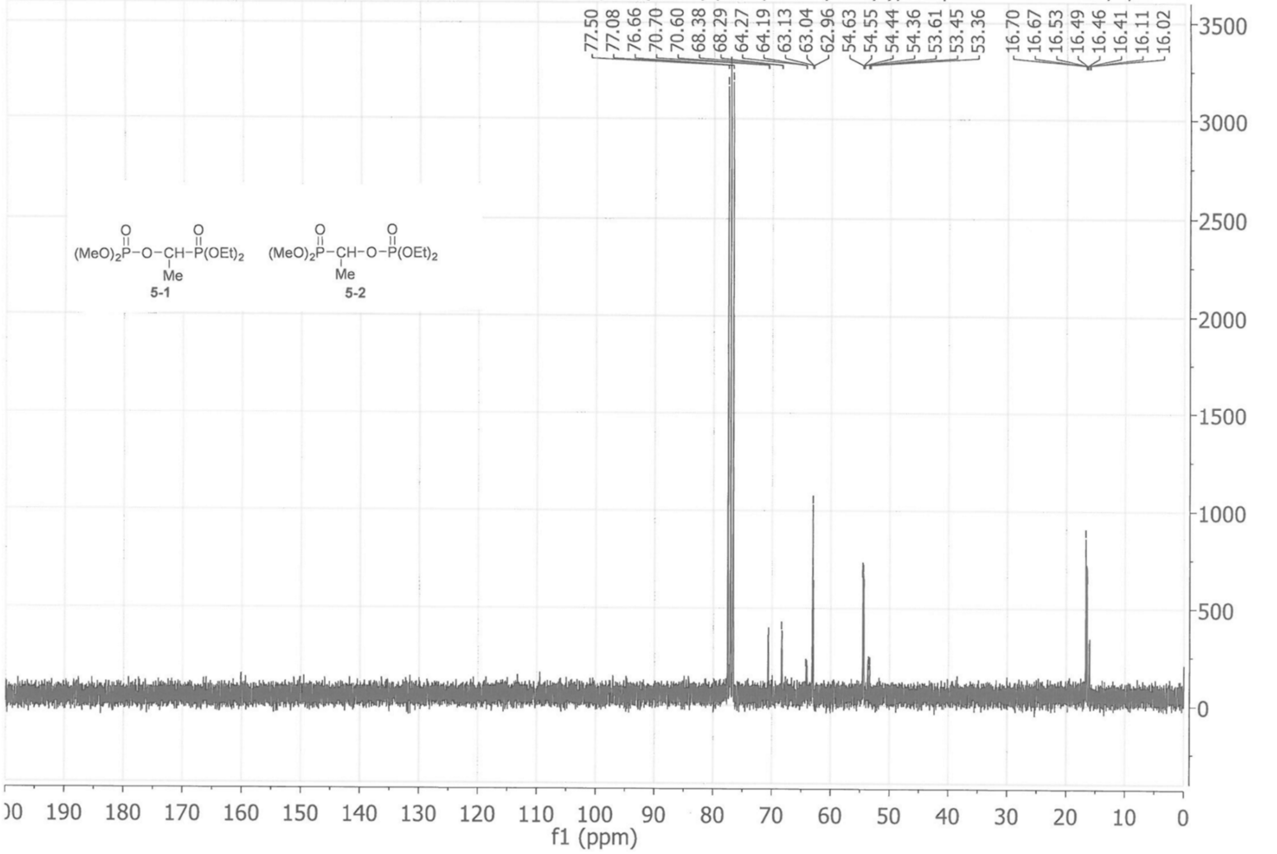

Diethyl 1-(dimethylphosphonoylethyl)phosphate and Dimethyl 1-(diethylphosphonoylethyl)phosphate SzZs43ism/2/3.fr. 1H 500M

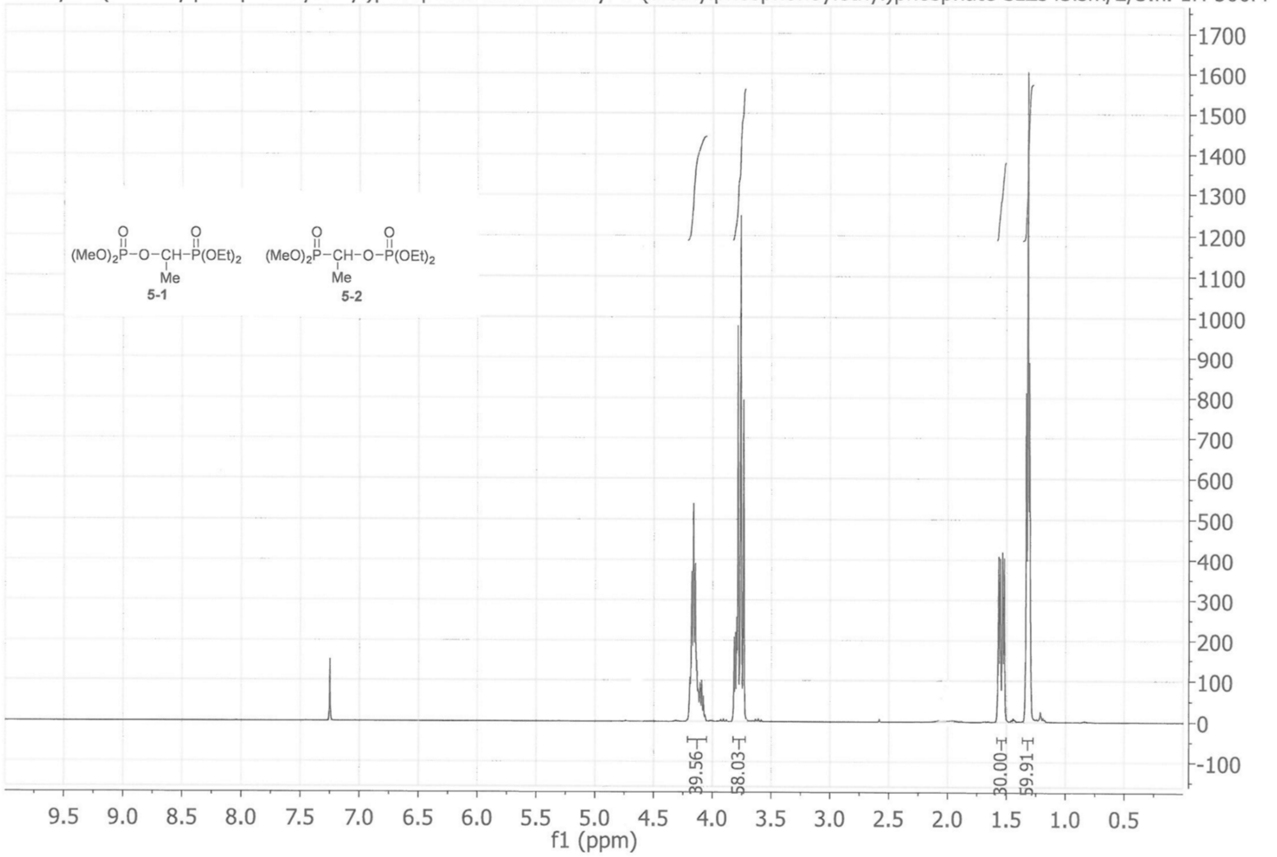

Diethyl (diethylphosphonoylbenzyl)phosphate SzZs70+72/3.fr. 31P 500MHz

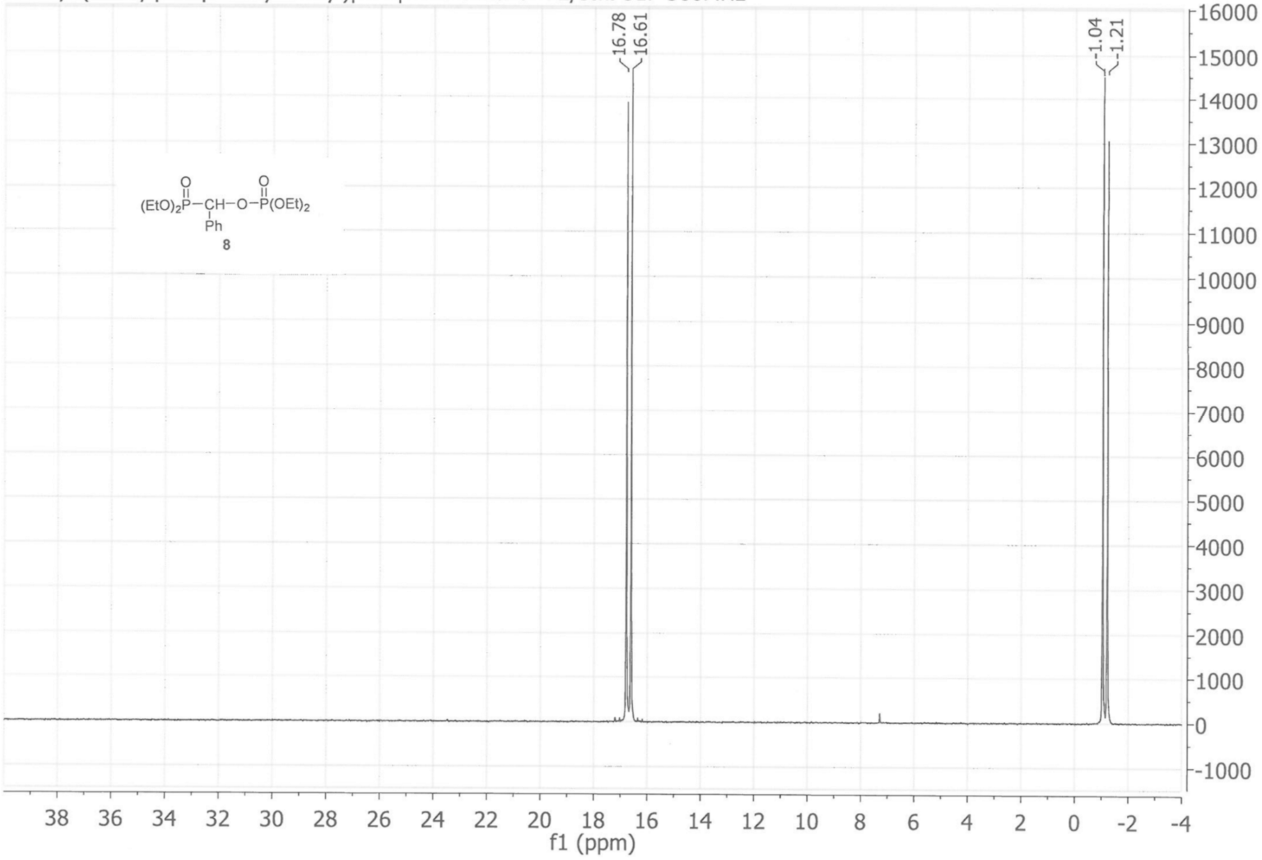

Diethyl (diethylphosphonoylbenzyl)phosphate SzZs70+72/2.fr. 13C 500MHz

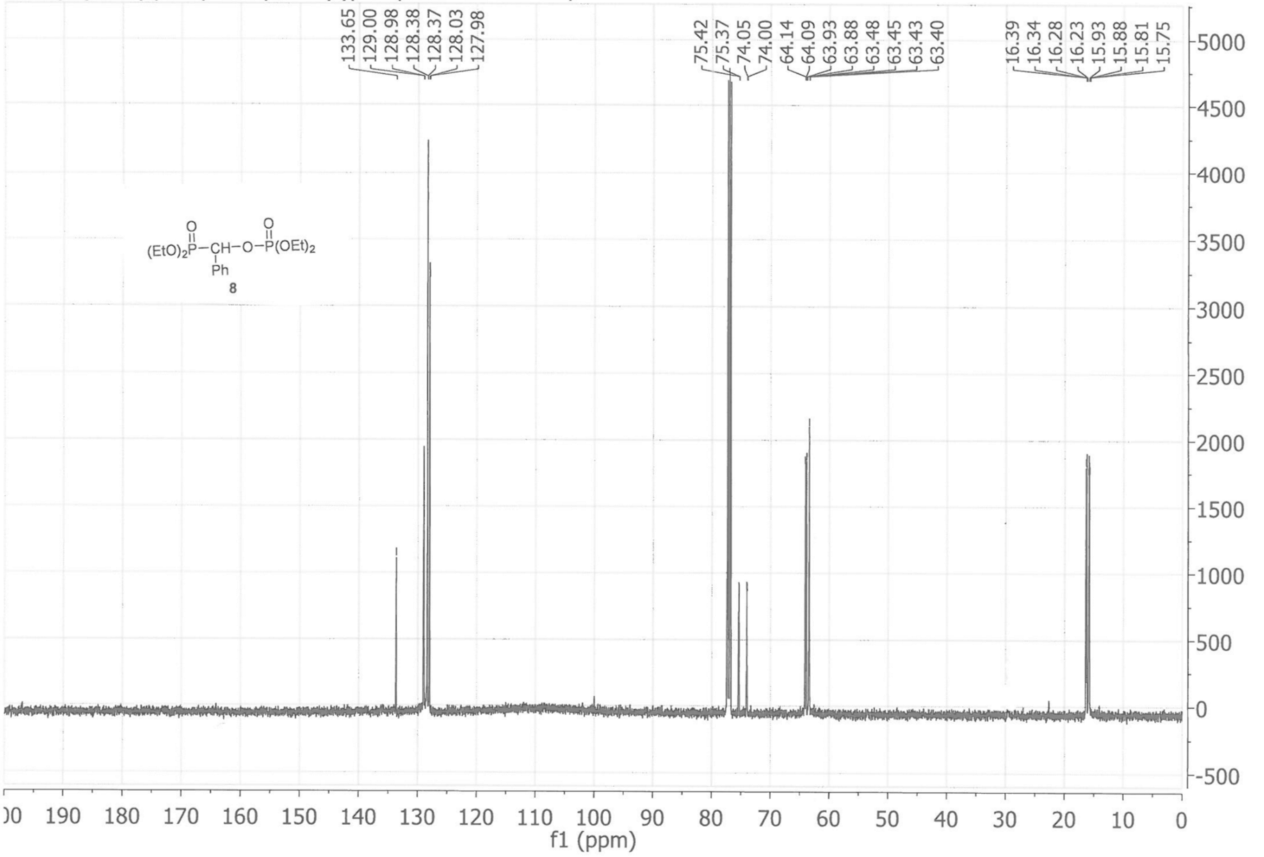

Diethyl (diethylphosphonoylbenzyl)phosphate SzZs70+72/3.fr. 1H 500MHz

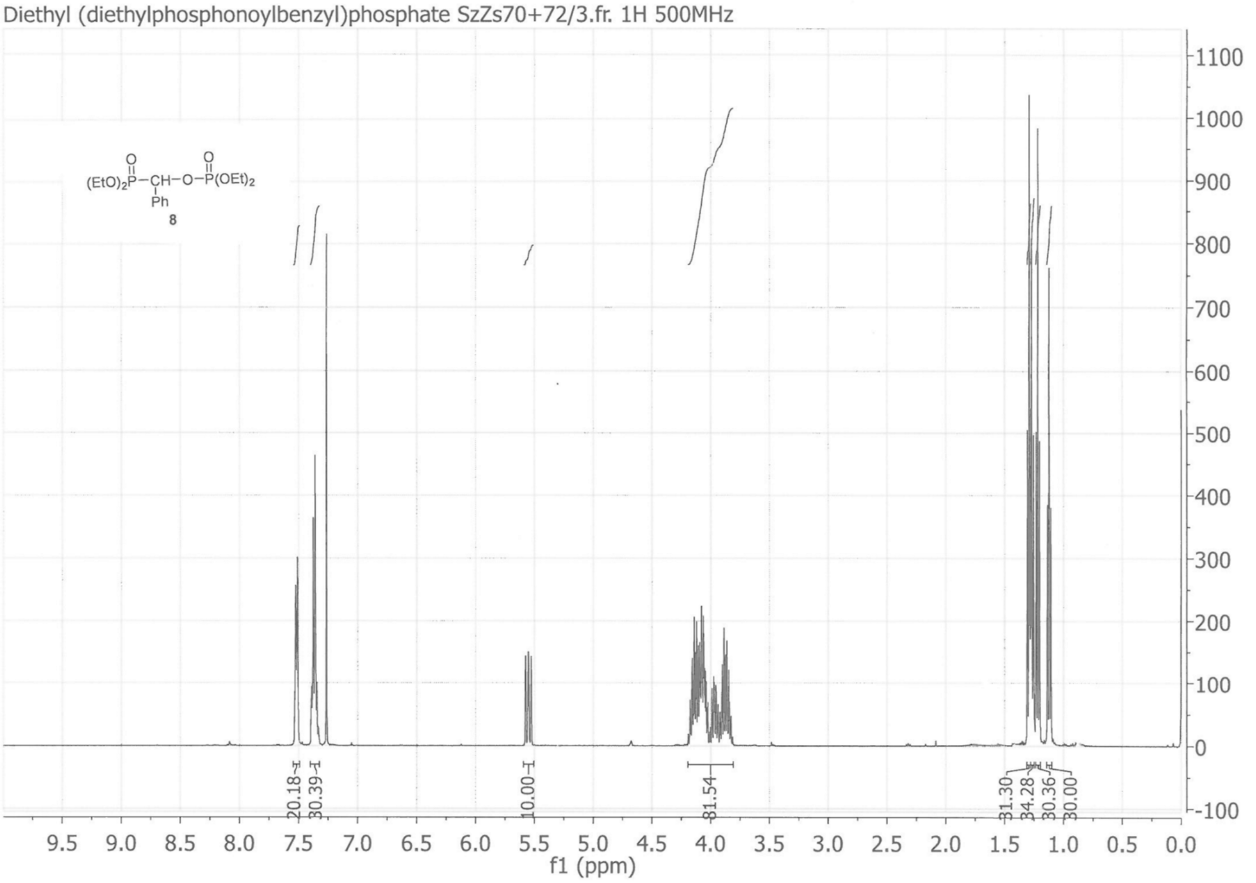

Diethyl (dimethylphosphonoylbenzyl)phosphate and Dimethyl (diethylphosphonoylbenzyl)phosphate SzZs74+75/II.o./2.fr. 31P 5[illegible]

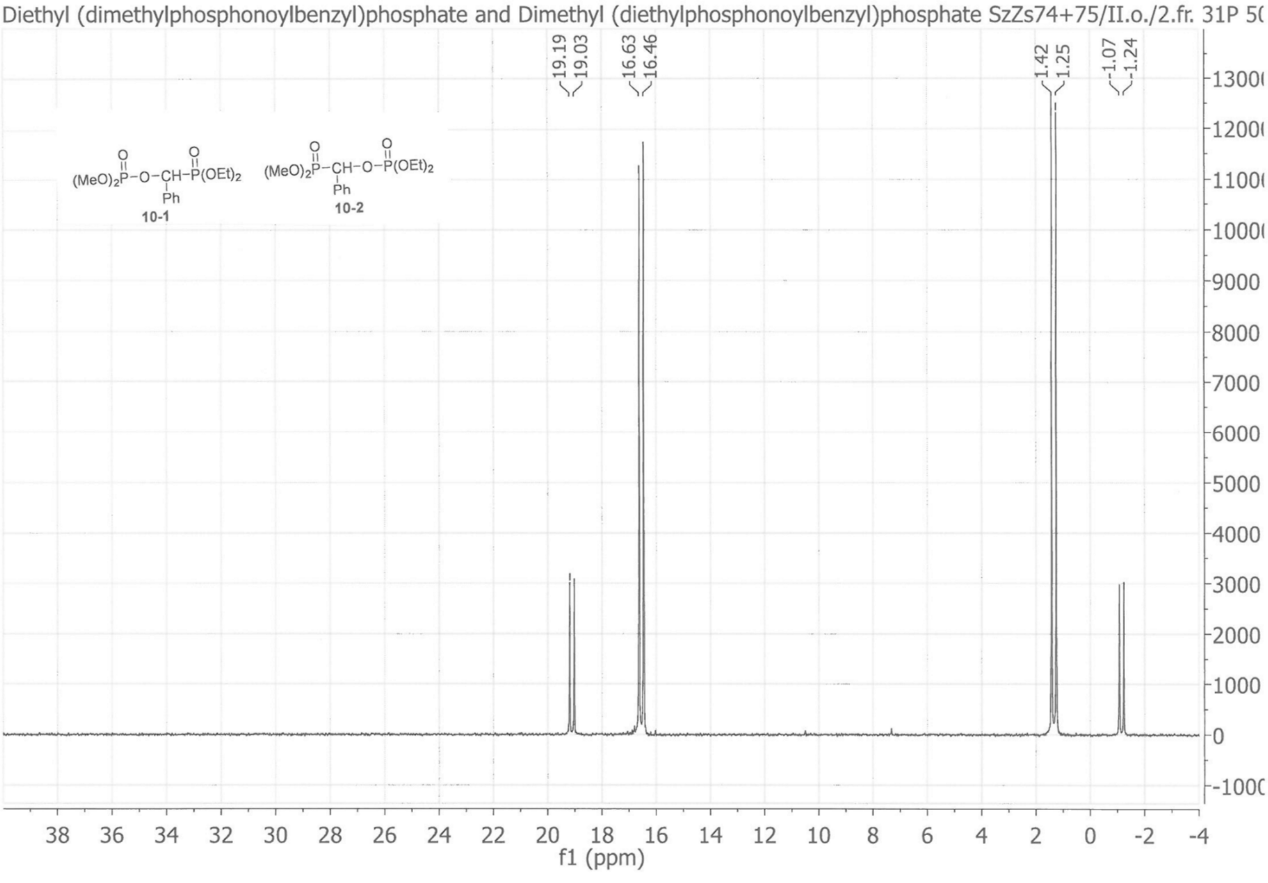

Diethyl (dimethylphosphonoylbenzyl)phosphate and Dimethyl (diethylphosphonoylbenzyl)phosphate SzZs74+75/II.o./2.fr. 13C 5(

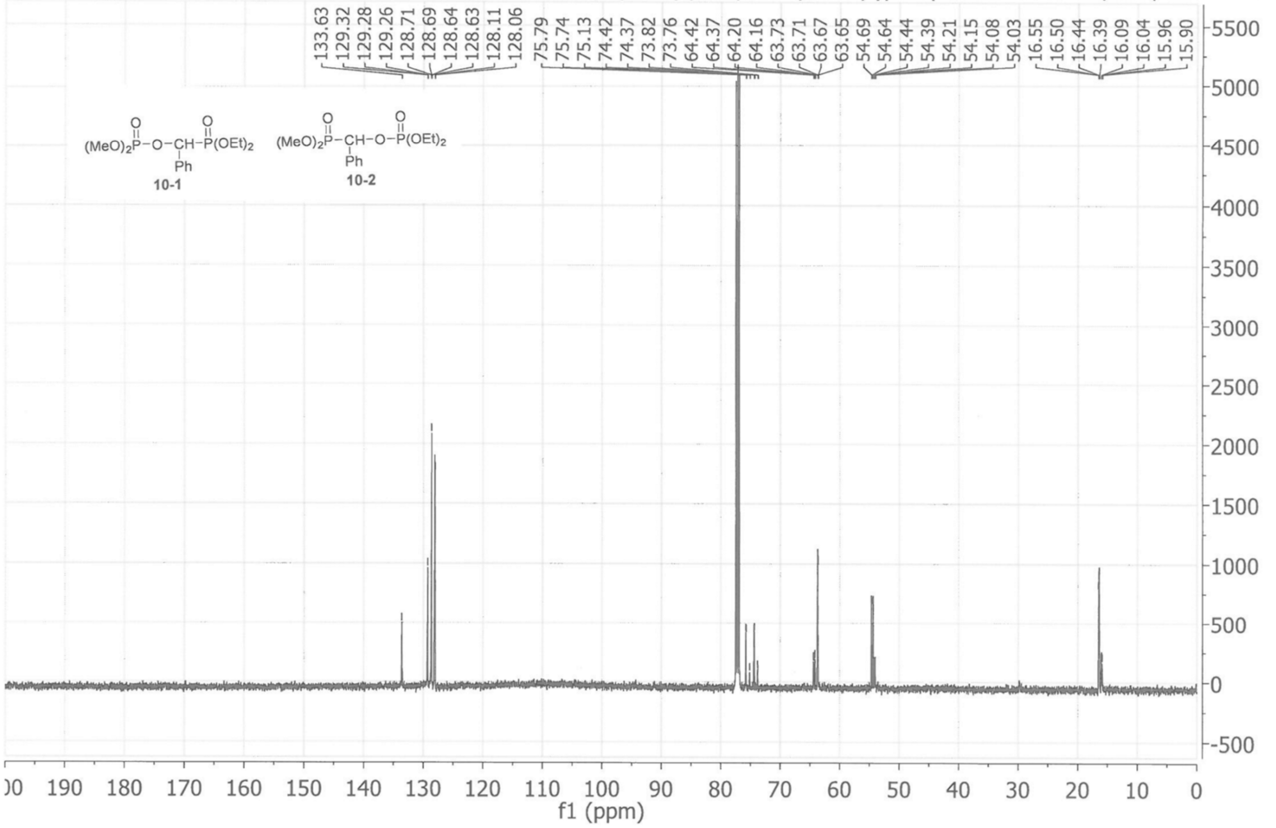

Diethyl (dimethylphosphonoylbenzyl)phosphate and Dimethyl (diethylphosphonoylbenzyl)phosphate SzZs74+75/II.o./2.fr. 1H 50(

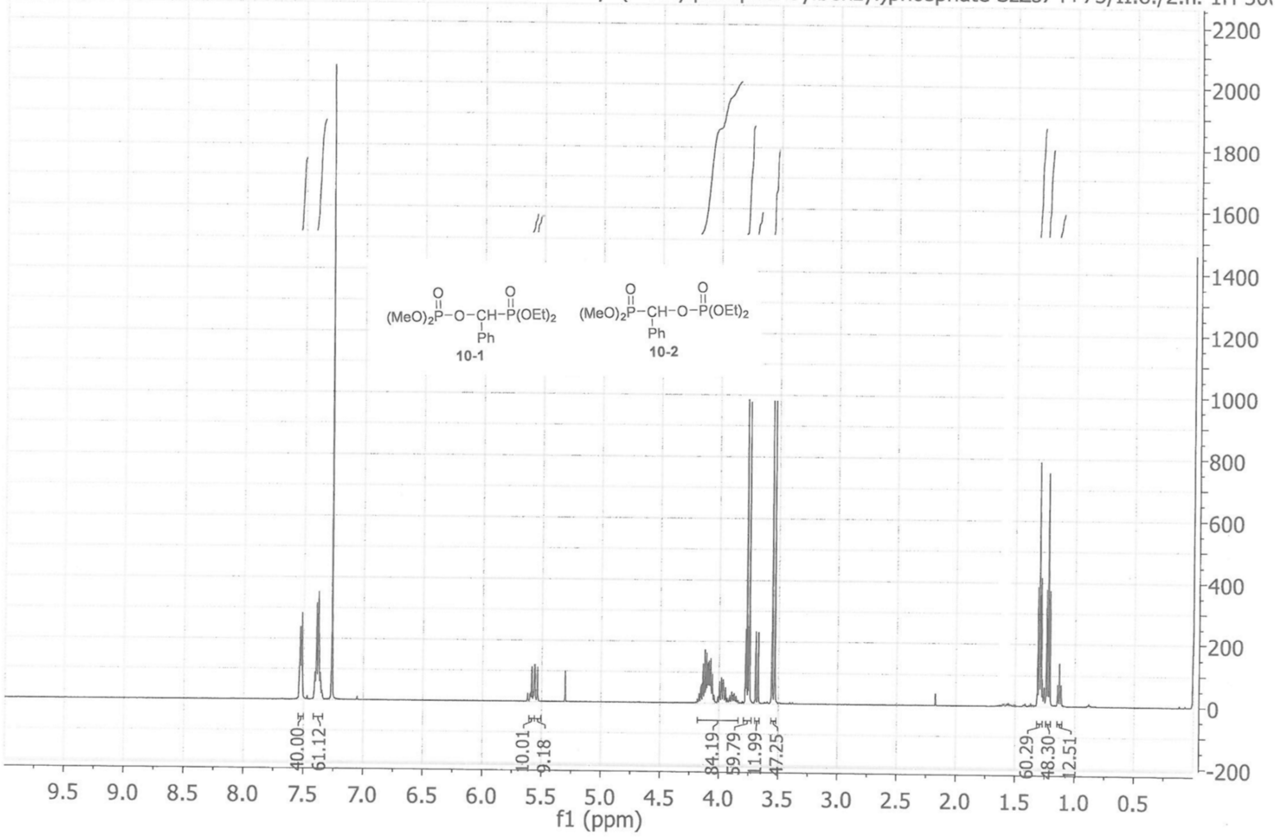

Diethyl 1-diphenylphosphinoyl-1-hydroxy-ethylphosphonate SzZs69+78+79/III.o./5.fr. 31P 500MHz

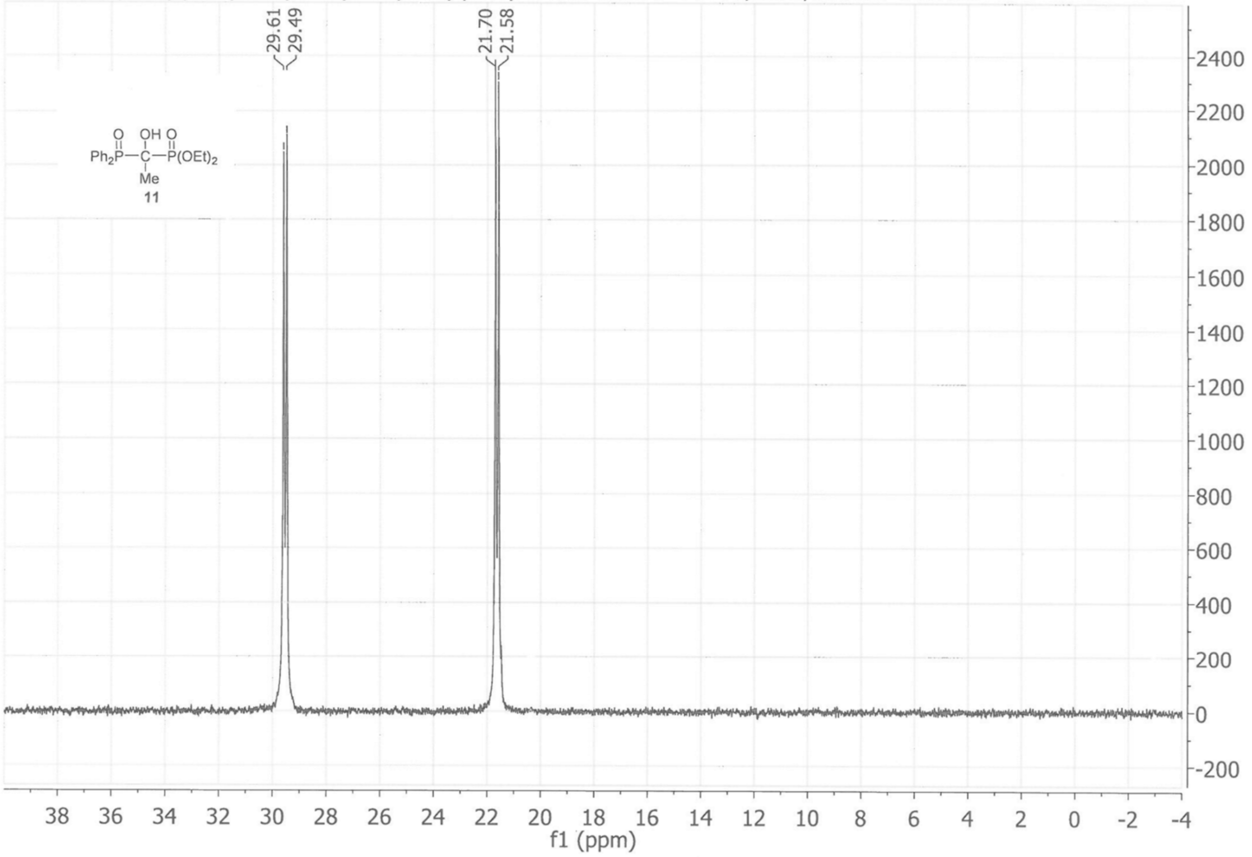

Diethyl 1-diphenylphosphinoyl-1-hydroxy-ethylphosphonate SzZs69+78+79/III.o./3.fr. 13C 300MHz

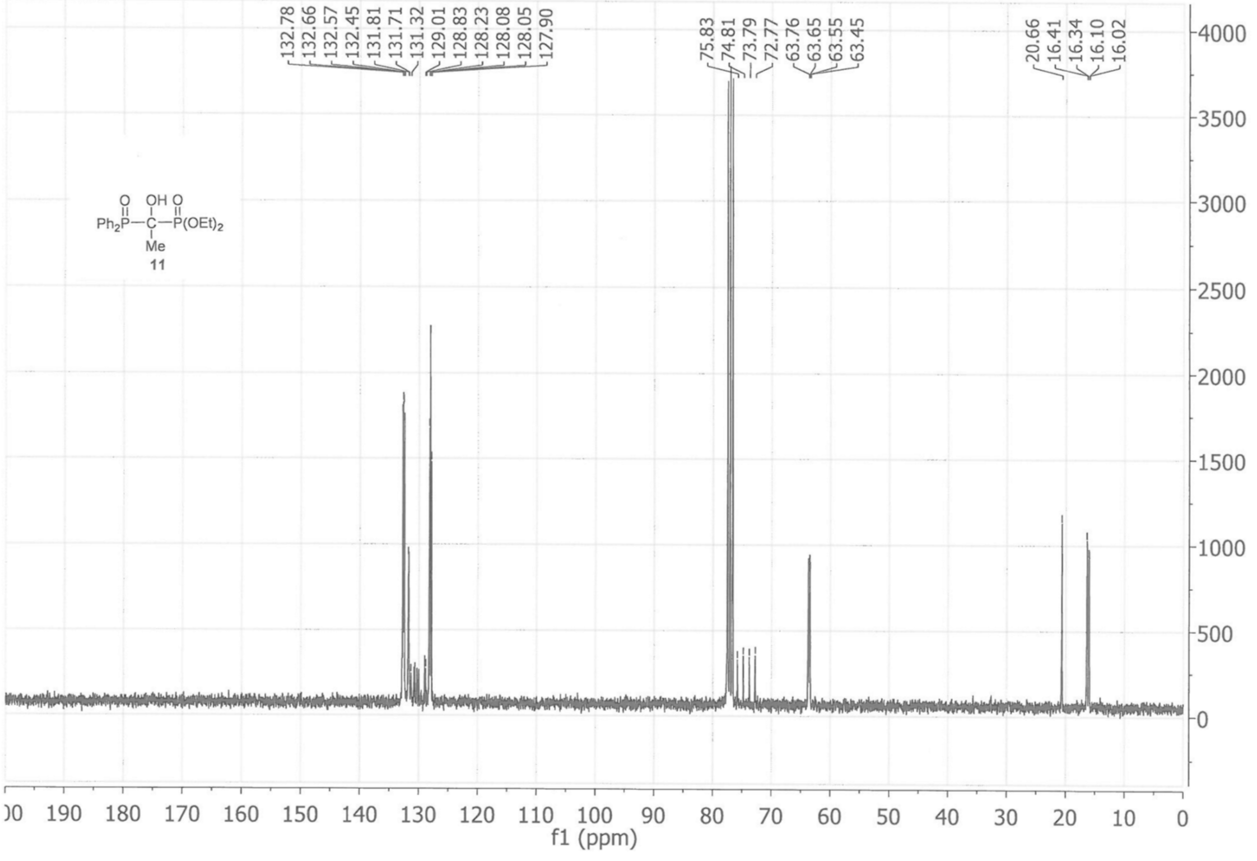

Diethyl 1-diphenylphosphinoyl-1-hydroxy-ethylphosphonate SzZs84átkr. 1H 500MHz

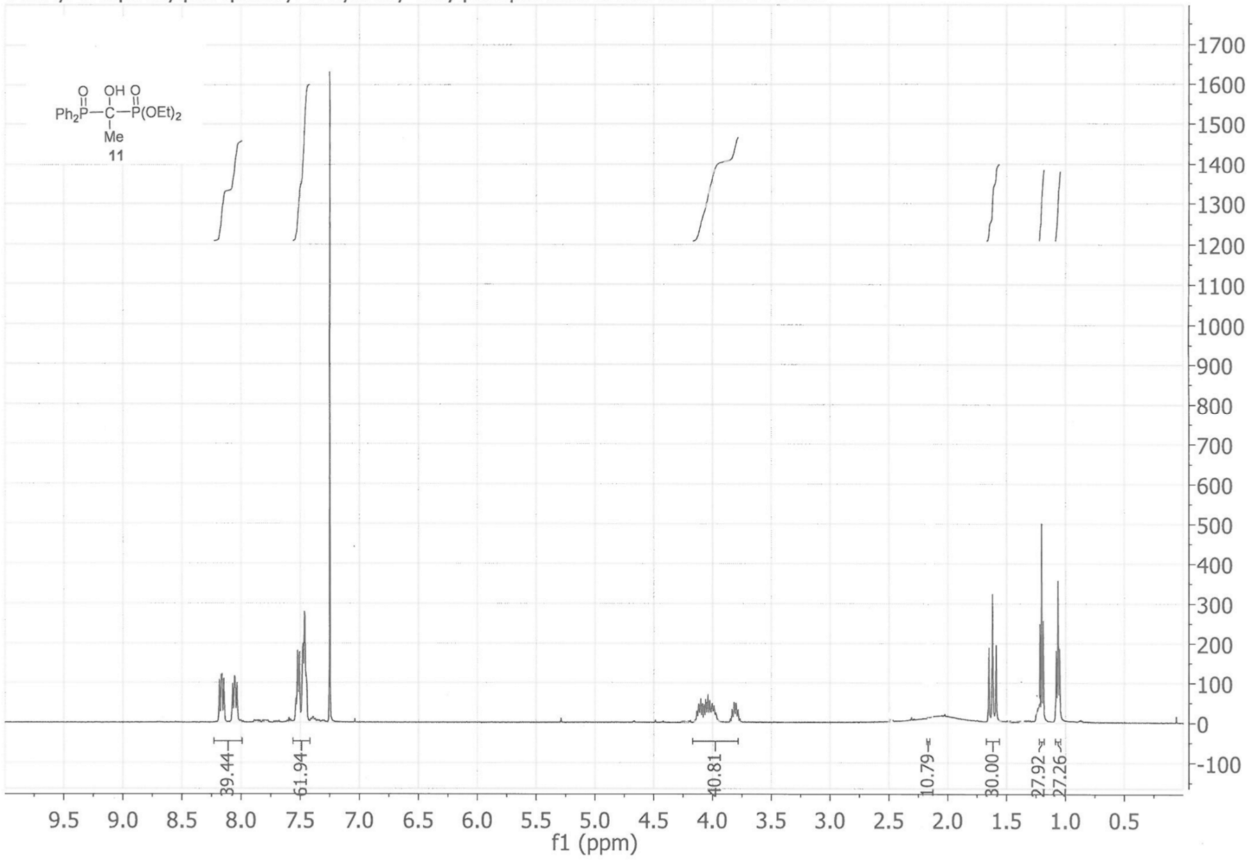

Diethyl (diphenylphosphinoylbenzyl)phosphate and Diethyl (diphenylphosphinoyloxybenzyl)phosphonate SzZs83/II.o./7.fr. 31P 5[illegible]

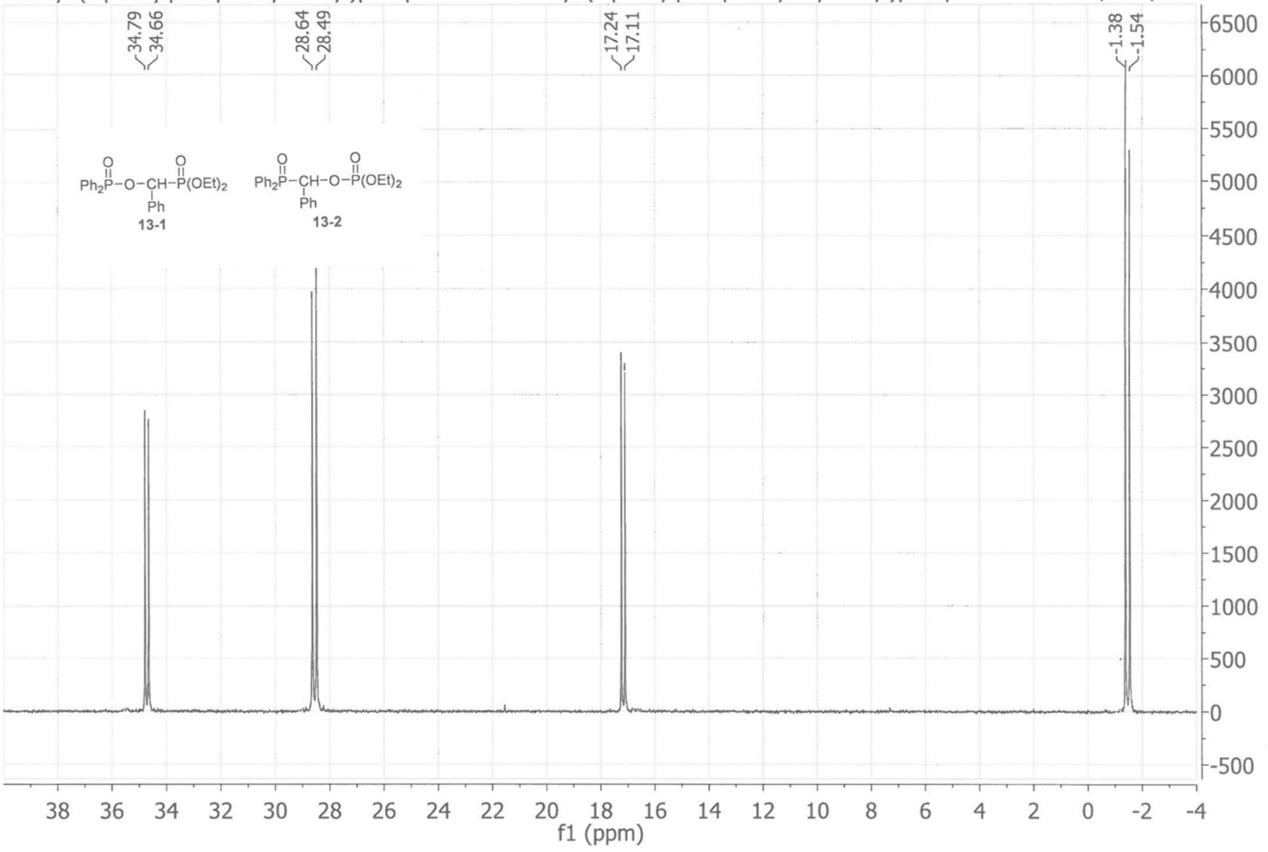

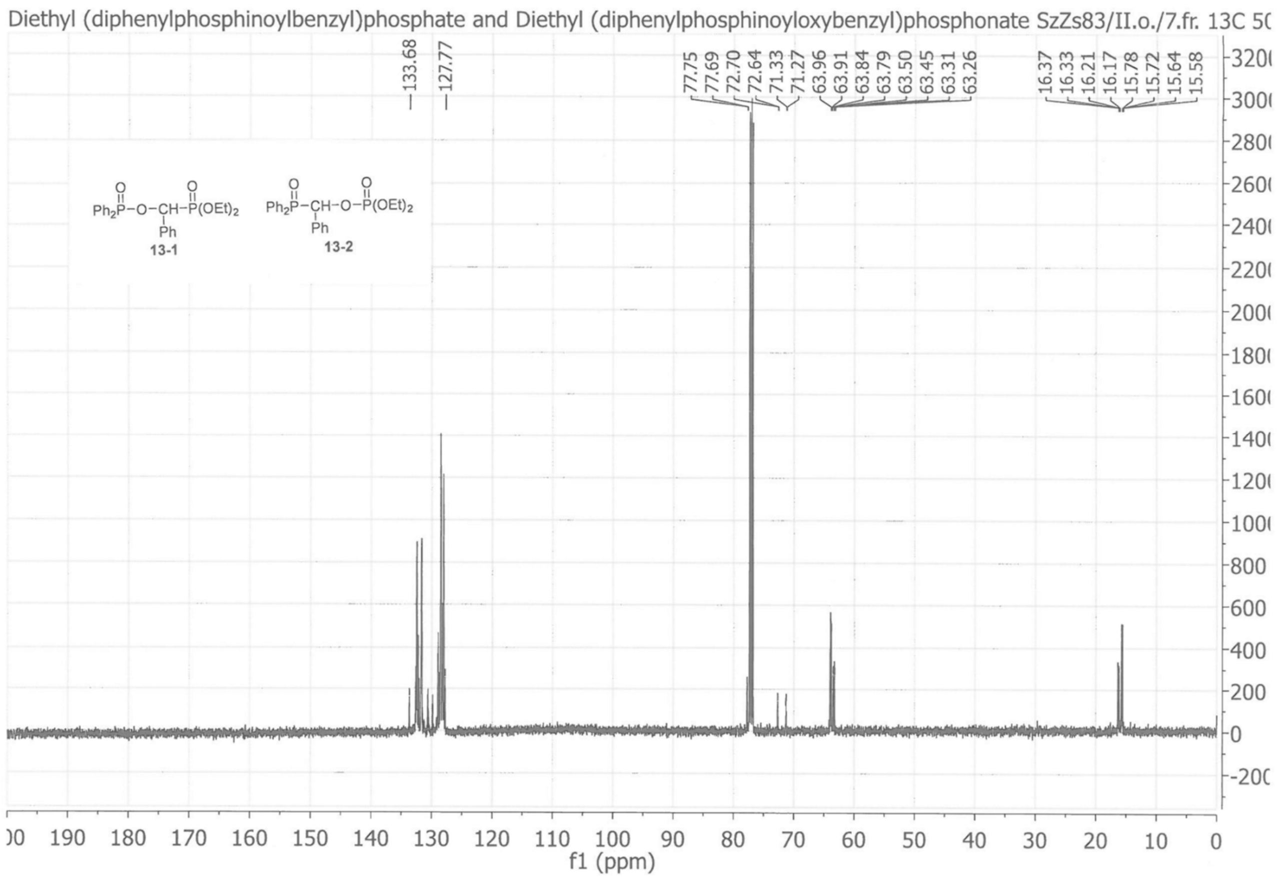
Diethyl (diphenylphosphinoylbenzyl)phosphate and Diethyl (diphenylphosphinoyloxybenzyl)phosphonate SzZs83/II.o./7.fr. 13C 50
133.68
127.77
77.75
77.69
72.70
72.64
71.33
71.27
63.96
63.91
63.84
63.79
63.50
63.45
63.31
63.26
16.37
16.33
16.21
16.17
15.78
15.72
15.64
15.58
13-1
13-2
f1 (ppm)

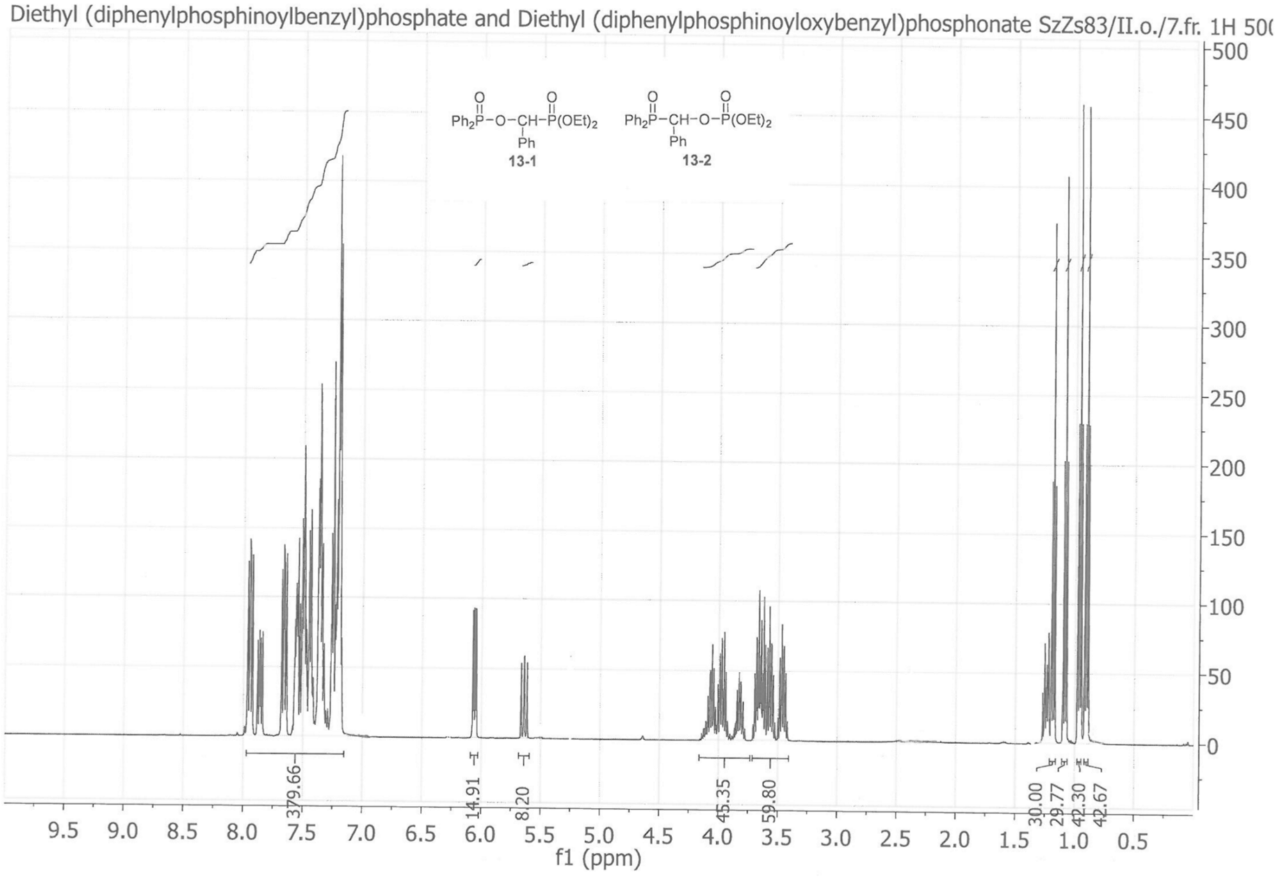
Diethyl (diphenylphosphinoylbenzyl)phosphate and Diethyl (diphenylphosphinoyloxybenzyl)phosphonate SzZs83/II.o./7.fr. 1H 50
13-1
13-2
379.66
14.91
8.20
45.35
59.80
30.00
29.77
42.30
42.67
f1 (ppm)
